# A systematic map of evidence on the relationship between agricultural production and biodiversity in tropical rainforest areas

**DOI:** 10.1186/s13750-024-00339-0

**Published:** 2024-06-02

**Authors:** Via Apriyani, Mukhlish JM Holle, Sonny Mumbunan

**Affiliations:** 1 Center for Climate and Sustainable Finance (CCSF), Institute for Sustainable Earth and Resources, University of Indonesia, Depok, Indonesia; 2 Department of Tropical Biology, Faculty of Biology, Gadjah Mada University, Yogyakarta, Indonesia; 3 Faculty of Social Sciences, Indonesian International Islamic University (UIII), Depok, Indonesia

**Keywords:** Rainforest, Tropical agriculture, Agroecosystem management, Farming practices, Aboveground biodiversity, Belowground biodiversity, Natural habitat

## Abstract

**Background:**

The tropical rainforest biome plays a significant role in providing habitats for terrestrial biodiversity and delivering ecosystem service values, contributing to agricultural production. However, the increasing demand for tropical commodities with high economic value threatens this humid ecosystem and its biodiversity. To our knowledge, no studies have systematically mapped the relationship between the impacts of agricultural production on biodiversity and the effects of biodiversity on agricultural production in tropical rainforest areas.

**Methods:**

Since we were interested in systematically mapping the evidence measuring the impact of tropical agriculture on biodiversity (Map 1), and the vice versa relations, the influence of biodiversity on tropical agriculture production (Map 2), we developed a respective set of search strings, eligibility criteria, and subsequently performed independent searching, screening, and data coding processes. We searched articles from six peer-reviewed databases and 22 gray literature sources. Articles were screened based on the inclusion criteria at the title, abstract, and full-text levels. Individual articles that passed full-text screening were coded and synthesized to create heatmaps. Selected information of interest was also extracted and visualized in the graphics which were clustered based on the year of publication, geographical distribution, type of rainforest, exposure, outcome, farm commodity, and study comparators.

**Review findings:**

Two heatmaps were generated from a contrasting number of references, with heatmap 1 extracted from 222 studies and heatmap 2 derived from 10 times fewer references (*n* = 20). In heatmap 1, impacts of land conversion to aboveground biodiversity and wild species and ecosystem functions in natural ecosystems were the most common relationships examined, with 115 articles and 62 articles, respectively. Conversely, heatmap 2 showed evidence that focused predominantly on the examination of the links between the impacts of genetic resource diversity on environmental factors and soil management in tropical agricultural production, with four articles each exploring these relations.

**Conclusions:**

These systematic maps reveal that while studies investigating the impacts of tropical agricultural production on biodiversity were abundant, studies examining the impacts of biodiversity on tropical agricultural production were lacking despite both systematic maps experiencing an increasing trend of publication during 2000–2020. Map 1 emphasized the examination of the effects of land conversion on aboveground biodiversity, and on wild species and ecosystem functions. Map 2 highlighted the influence of crop genetic resources on environmental factors, and on soil management as the most frequently studied. The evidence cluster identified here can be the starting point for further systematic review study (to assess, for example, their cause–effect significance).

**Supplementary Information:**

The online version contains supplementary material available at 10.1186/s13750-024-00339-0.

## Background

Tropical rainforest biomes, although occupying only about 18% of the Earth’s total land area [1] or 7% of its total surface area, play a significant role on a global scale [2]. Situated in the equatorial zone, tropical rainforests provide habitats for terrestrial biodiversity, encompassing approximately 72% of birds, 63% of mammals, and 76% of amphibians [1] globally. Moreover, the rich biodiversity in tropical rainforests offers various ecosystem services crucial for regulating climate (i.e., carbon sequestration), supporting biogeochemical or nutrient cycles, and maintaining ecosystem resilience [3, 4].

### Impact of biodiversity on agricultural productivity

Ecosystem services provided by tropical biodiversity, such as seed dispersal, pollination, and pest control in agroecosystems and natural ecosystems, are important for global food supply [3, 5]. For example, 70% of the world’s most important crop commodities rely on animal pollinators for fruit formation [6]. The economic value of pollination services was estimated to range from US$195 billion to approximately US$387 billion in 2020 [7, 8]. Hence, the loss of pollination services could lead to crop failures and subsequent food scarcity [9]. Additionally, tropical crop production, supported by ecosystem services, contributes to income generation and economic growth through crop commodity trade [10].

### Impacts of agricultural production on biodiversity

In addition to generating economic revenues, tropical agriculture commodities threaten the extent of tropical rainforests and the biodiversity they contain [11], particularly evident in developing countries where many tropical rainforests are located [12]. Forest conversion and farm intensification have resulted in forest and habitat loss [13], impairing biodiversity hotspots [14]. During the 1990s, the average loss of tropical forests recorded ranged from 50,000 to 120,000 km^2^ per year [15], which was particularly significant in the Brazilian Amazon and Tropical Asia [16]. Losses of tropical forest cover further place biodiversity hotspots on the verge of extinction, despite many of these endemic hotspots retaining exceptional terrestrial vertebrates and vascular plants [15].

Agriculture can support biodiversity by adopting specific farming practices, such as polyculture and agroforestry. Additionally, preserving forested areas within agricultural landscapes can help sustain wildlife habitats and populations [17]. Agroforestry and mixed farming systems, comprising various crop plants and complex tree layers, can serve as habitats for insects, birds, soil biota, and other native animals and plants [18]. Fragmentation resulting from forest conversion to cropland can create patches that, if ecologically well managed (e.g., providing corridors and maintaining buffer vegetation), can become habitats and foraging areas for forest species [12, 17, 18].

Furthermore, tropical agriculture management determines whether agriculture is biodiversity friendly, ultimately determining the ecosystem services that biodiversity can provide to agriculture. Numerous primary studies have been conducted to understand the relationship between agriculture production and biodiversity [19, 20] and vice versa [21–23]. However, to our knowledge, no studies have systematically mapped the impact of tropical agricultural production on biodiversity, or vice versa, the impact of biodiversity on agriculture in tropical rainforest areas. Therefore, we aim to quantify the distribution of existing studies on the impacts of tropical agricultural production and farm management on biodiversity in agroecosystems and natural ecosystems, and vice versa relations. This systematic mapping reveals the temporal and geographical trends of the impacts of tropical agricultural production and farm management on biodiversity, and vice versa. Additionally, our mapping uniquely offers two independents unidirectional relationships that equally recognize the importance of tropical agriculture management and the role of biodiversity on a multispatial scale.

**Fig. 1:**
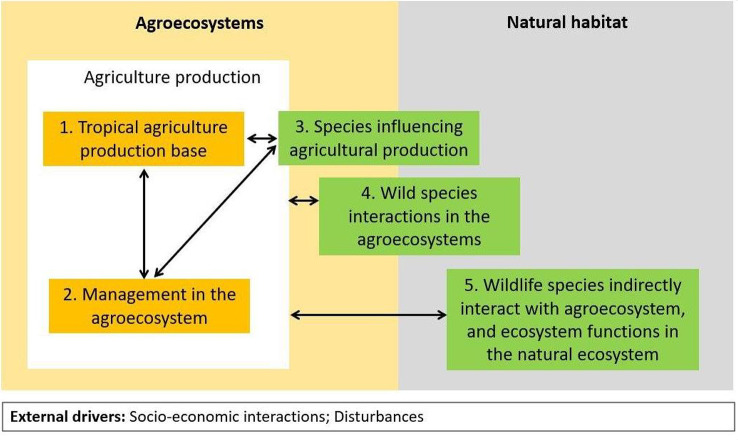
Schematic diagram of the Tropical Agriculture-Biodiversity Framework (TABF). *Note*: Orange boxes refer to agriculture production components and green boxes refer to biodiversity component

A context-specific framework is required to understand the relationship pathways through which agriculture and biodiversity influence each other, particularly in tropical rainforest areas. We have developed a framework called the Tropical Agriculture and Biodiversity Framework (TABF), adapted from a well-established agri-biodiversity framework developed by the Organization for Economic Cooperation and Development in 2001 [24]. TABF includes biodiversity and agricultural elements and a precise spatial division between agroecosystems and natural ecosystems tailored to tropical rainforest situations. The framework also has a clear spatial classification of different biodiversity components (species influencing agricultural production living in agroecosystem, species coexisting in the agroecosystem, and species outside of the agroecosystem) and covers different roles of biodiversity in agricultural production that may involve either ecosystem services or disservices. The five main indicators illustrated in the TABF are as follows: (1) tropical agriculture production base, (2) management of the agroecosystem, (3) species influencing agricultural production, (4) wild species interactions in agroecosystems, and (5) wild species and ecosystem functions in natural ecosystems. The TABF is shown schematically in Fig. 1 below.

1

The TABF offers a framework specific to tropical agriculture that is relevant to the wide range of tropical crops. For example, the sub-indicator crop diversification (under the indicator number 2) acknowledges the variety of agroecosystem management practices, as some tropical crops are mostly monoculture (e.g., rice, oil palm, and maize) while others can be managed as part of an intercropping system.

### Objective of the review

In this systematic mapping study, we primarily aimed to map the quantity and distribution of existing studies that evaluated the impacts of tropical agricultural production and farm management on biodiversity in agroecosystems and natural ecosystems. Additionally, we aimed to map articles measuring the role of tropical biodiversity in agricultural production and management, given the importance of these topics. To our knowledge, such a systematic mapping study has not yet been developed. The demonstrated maps and findings from this study allow us to assess which relationships between agricultural practices and tropical biodiversity have been studied the most and which relationships require greater attention as research priorities in the future. Therefore, we addressed the following primary research question:

### What evidence exists on how tropical agricultural production activities and biodiversity influence one another?

This question was formulated by researchers across disciplinary studies involved in this research and has been discussed and reviewed by senior experts in the fields of agriculture, ecology, and conservation biology through a series of online webinars and discussions to ensure its significance is pressing enough to be examined.

Given the primary research question, we developed the following set of secondary research questions:

1. *What is the state of evidence for tropical agricultural production and its relationship with biodiversity regarding the quantity of articles, study types, commodity types, and geographical locations?*
2. *What evidence exists regarding the impacts of tropical agricultural production bases and agroecosystem management on biodiversity?*
3. *What evidence exists regarding the impacts of biodiversity on tropical agricultural production?*
4. *What are the major knowledge gaps in the evidence base that warrant future research priorities?*

As we intended to create two evidence maps illustrating the two independent unidirectional relationships between tropical agriculture and biodiversity, we defined the following two sets of key elements based on the second and third secondary research questions:

### What evidence exists regarding the impacts of tropical agricultural production bases and agroecosystem management on biodiversity?

#### Population

Tropical rainforest areas.

#### Exposure

Tropical agriculture production base and management of the agroecosystem.

#### Comparator

Spatial comparator in empirical or experimental studies comprises farmland with or without the agricultural interventions, and studies comparing natural or secondary forests versus farms with the interventions. Furthermore, studies comparing similar types of crop agriculture in different locations (e.g., fields, regions, or countries) will be considered. Temporal comparators examine the difference in outcomes before and after an agricultural intervention or natural disturbance exposure or compare exposures at different points in time (e.g., seasonal changes). The comparisons include different agroecosystem management methods (comparators within group of exposure) applied by farmers. For instance, comparators within groups in the exposure type of soil management are soil tillage versus chemical fertilizer amendment, in the crop diversification exposure type are polyculture with timber versus spice trees, and in the pest management exposure are pest management with natural fallows versus planted fallows, or use of different types of herbicides, and insecticide, etc.).

#### Outcome

Changes in biodiversity indicators (e.g., species richness, abundance, composition, and density) in agroecosystems and natural ecosystems.

### What evidence exists regarding the impacts of biodiversity on tropical agricultural production?

#### Population

Agriculture in tropical rainforest countries producing priority crop commodities.

#### Exposure

Existence of biodiversity (e.g., species richness, abundance, composition, and density) and mediated functionality (i.e., ecosystem services/disservices) in agroecosystems.

#### Comparator

Spatial comparator includes agroecosystems across different spatial locations. Temporal comparators involved comparing exposures at different points in time. Within the group of exposures, the comparators were agroecosystems without, or with (either less or more) biodiversity mediating functionality.

#### Outcome

Changes in tropical agricultural production base (i.e., availability of edaphic and climatic factors and availability and suitability of crop varieties) and agroecosystem management suitability (i.e., management of soil, water, pest, and crop diversification).

## Methods

This systematic mapping study adhered to a previously published protocol in Environmental Evidence [25] that followed the Guidelines and Standards for Evidence Synthesis in Environmental Management [26]. This systematic mapping study followed the RepOrting standards for Systematic Evidence Syntheses (ROSES) [27] (Additional File 1). The following sections explain our steps in systematically mapping the relationships between tropical agriculture and biodiversity, and vice versa, including a slight deviation from the earlier protocol.

### Deviations from the protocol

After evaluating the search results from the search strings listed in the protocol [25], we updated the strings used for Maps 1 and 2. In Map 1, we added several terms, such as “smallhold*” OR “small-hold*” OR “small-scale” OR “large hold*” OR “large-hold*” OR “large-scale” OR “natural disturbance*” OR “volcanic eruption*” OR “hurricane*” OR “outbreak*.” These additional terms, particularly the last four, are important inclusions, as they pertain to one of the exposure types specified in our inclusion criteria.

Similarly, in Map 2, we added synonyms of the outcome terms “small-scale” OR “small scale” OR “smallhold*” OR “large-scale” OR “large scale” OR “large hold*” OR ”industr*”. Adding these terms helped us to expand the scope of the search results, considering that the terms used in the articles might have come from various disciplines (e.g., agriculture and biological sciences from natural or social science perspectives). We applied the updated and more comprehensive search strings to search for citations on several peer-reviewed databases since the search functions in each peer-reviewed source varied and the gray literature databases allowed only limited search strings. Entering the full search strings was not possible in Science Publishing Group, Directory of Open Access Journals, and AGRICOLA databases as these sources had a maximum number of key search terms. An updated search in the AGRIS database was not performed since we did not have access anymore. Thus, the search using updated strings was performed in Scopus and Web of Science Core Collection. However, due to the large number and irrelevant hits discovered by the Web of Science and given the limited time and resources, we decided to process the updated search results from Scopus only. The results obtained from these updated strings in Map 1 and Map 2 were subsequently screened at the title, abstract, and full-text levels and extracted once they met all the inclusion criteria.

Additionally, we modified the coding sheet used to code extracted information in Map 1 based on feedback from reviewers on the exposure and outcome types provided in the protocol paper. The outcome types of environmental factors and genetic resources were removed to focus on the outcome types of the aboveground and belowground biodiversity aspects.

We also recorded the design comparator i.e., existence of comparator within groups and type of comparator used in the articles. The comparator comprises spatial and temporal comparators. In Map 1, the spatial comparator encompasses farmland with or without the agricultural interventions, and studies comparing natural or secondary forests versus farms with the interventions, including comparators within groups of exposure (different agroecosystem management methods) and within different locations (e.g., fields, regions, or countries). The spatial comparator in Map 2 includes agroecosystems across different spatial locations. Within the group of exposures, the comparators were agroecosystems without, or with varying levels (either less or more) of biodiversity mediating functionality.

Temporal comparators in Map 1 and Map 2 examine the difference in outcomes before and after an agricultural intervention or natural disturbance exposure or compare exposures at different points in time (e.g., seasonal changes). Articles that have a comparator but not the comparator within the group of exposure type we initially defined as eligibility criteria will be coded as ‘studies with other types of comparators’.

In an attempt to increase the consistency and reliability of the first stage of screening results, in the protocol, we planned to assign two reviewers to screen independently the title and abstract levels. However, given the large number of total citations, we decided to review each title and abstract by one reviewer only. In case of hesitation, whether to include or exclude, we tend toward inclusion at this stage. To check consistency, we assigned two reviewers to screen 6.9% of the total 11,538 citations. Any disagreement on the screening results at this stage was discussed before the subsequent screening process was started.

In terms of duplicate checking, in the protocol, we expected that Colandr would remove the duplicates automatically. However, since numerous similar citations were captured and obtained from different databases, non-identicality due to different punctuation marks (e.g., period, dash, colon, question mark, etc.) on titles of the same articles occurred and caused the inability of Colandr to detect duplicates. Instead of using Colandr, we spotted duplications before the first stage screening through Microsoft Excel feature (‘Conditional Formatting’ > ‘Highlight Cell Rules’ > ‘Duplicate Values’) and at the full-text screening manually through scanning of the title. Before duplicate checking in Microsoft Excel was performed, we ensured that all titles were identical and did not have any different punctuation marks. Finally, the exclusion of duplicates at the full-text screening was recorded as “Double” in the ROSES flow diagram.

### Search for articles

#### Search terms and strings

The search strings for Maps 1 and Maps 2 were developed and reviewed by a research team from diverse disciplinary backgrounds, including biology, environmental and sustainability science, social science, and economics. We formulated the strings using primary terms related to population, exposure, and outcome, along with their synonyms, combined through Boolean operators and syntax features (e.g., wildcards, truncation, double quotation marks, and lemmatization). Since the subjects of relations being investigated in systematic map 1 (impacts of tropical agricultural production to biodiversity) was an inverse of systematic map 2, we used exposure terms in map 1 as outcome terms in map 2, and the other way around, outcome terms in Map 1 as exposure terms in Map 2. Few additions on the operational terms were applied dependent on the exposure and outcome terms being scoped in this study.

Below are the updated strings used to search articlesin the Scopus database of systematic map 1 and map 2. Search fields in Scopus include article title, abstract, and keywords.

#### Map 1

rain forest” OR “rainforest” AND “tropic*” OR “humid” OR “moist” OR “equator*” AND “agri*” OR “agro*” OR “farm*” OR “crop*” OR “horticulture” OR “food produc*” OR “cultivat*” OR “yield produc*” OR “smallhold*” OR “small-hold*” OR “small-scale” OR “large hold*” OR “large-hold*” OR “large-scale” OR “natural disturbance*” OR “volcanic eruption*” OR “hurricane*” OR “outbreak*” AND “species” OR “wildlife” OR “plant” OR “plant*” OR “fauna” OR “flora” OR “animal” OR “insect” OR “insect*” OR “microb*” OR “microorgani*” OR “bacter*” OR “fung*” OR “invertebrate” OR “pollinat*” OR “mammal” OR “bird” OR “livestock” OR “invasi*” OR “divers*” OR “biodiversity” OR “group*” AND “rich” OR “rich*” OR “even*” OR “abundan*” OR “change” OR “dynamic” OR “functio*” OR “conflic*” OR ”interac*” OR ”distur*” OR ”alter*” OR ”decline” OR ”decrease” OR ”reduc*” OR ”loss” OR ”contraction” OR ”increase” OR ”gain” OR ”grow*” OR ”restor*” OR ”expansion” OR ”effec*” OR ”affec*” OR ”respon*” OR ”relatio*” OR ”influenc*.

#### Map 2

rain forest” OR ”rainforest” AND ”tropic*” OR ”humid” OR ”moist” OR ”equator*” AND ”role” OR ”effect” OR ”impact” OR ”contribution” OR ”function” OR ”relationship” AND ”species” OR ”wildlife” OR ”plant” OR ”plant*” OR ”fauna” OR ”flora” OR ”animal” OR ”insect” OR ”insect*” OR ”microb*” OR ”microorgani*” OR ”bacter*” OR ”fung*” OR ”invertebrate” OR ”pollinat*” OR ”mammal” OR ”bird” OR ”livestock” OR ”invasi*” OR ”divers*” OR ”biodiversity” OR ”group*” AND ”agri*” OR ”agro*” OR ”farm*” OR ”crop*” OR ”horticulture” OR “food produc*” OR ”cultivat*” OR “yield produc*” AND ”management” OR ”practice” OR ”soil” OR ”water” OR ”irrigation” OR ”pond” OR ”pest” OR ”pesticide” OR ”weed” OR “farming system” OR ”monoculture” OR ”polyculture” OR ”agroforest” OR ”intercrop*” OR “small-scale” OR “small scale” OR “smallhold*” OR “large-scale” OR “large scale” OR “large hold*” OR ”industr*.

Article searching was conducted twice, the first from January to February 2021 and the second in August 2021. The first round searching process was performed using the search strings registered in the protocol paper. During the search process, entering a full search string was not possible since each database has a different maximum number of allowable search terms. Therefore, in several databases, the strings were modified by shortening and only selecting the primary key terms of exposure and outcome.

The second search was conducted after we revised the search strings slightly, as explained in the deviations section above. The search was focused solely on Scopus and Web of Science Core Collection since the remaining peer-reviewed databases and the gray literature sources (e.g., organizational websites) have a maximum number of search terms that can be entered in the search function. For Map 1, Scopus gave 1,473 hits, and for Map 2, 517 hits were discovered. We compared these results with the results from the first round to avoid duplication. It resulted in the addition of 397 new and unique citations for Map 1, and 310 citations for Map 2. Web of Science Core Collection gave us a substantial number of citations, reaching 27,659,283 for Map 1 and 19,833,468 for Map 2. Compared to the first-round search results (1,764 for Map 1, and 306 for Map 2), these numbers seemed peculiar. Additionally, from our quick scanning, many of the citations resulting from the Web of Science were irrelevant. Thus, from the second round search, only citations from the Scopus databasewere subsequently processed to the screening stages.

#### Comprehensiveness of the search

Since we aimed to perform as comprehensive a search as possible, several measures have been carried out. First, we identified key search terms and their synonyms that reflect the keywords of population, exposures, and outcomes of this study. These terms were combined using Boolean operators to form a solid search string. The established strings were refined through iterative pilot testing in Scopus. Second, before applying the string in the search process, we assessed the sensitivity and tested its coverage in capturing relevant articles by running a test search and comparing the result against a set of test libraries (attached as Additional File 2 in the protocol paper [25]). The test libraries were provided by the research team based on their comprehension of the conceptual framework and eligibility criteria, and through scrutinizing bibliography lists from existing studies within the subject of interest. All citations in the test library (10 citations each in Map 1 and Map 2) were successfully captured using the string in our test search.

Moreover, a more comprehensive search string we used during the search process performed in August 2021 yielded the addition of original citations discovered, and eligible articles being extracted.

#### Search limitations

We limited the search to a certain range of years and the language used in the articles. Notably, we only accounted for articles published from 2000 to 2020. Furthermore, the search was conducted in 2021, and due to time and resource constraints, the terms and strings applied in the search functionalities were solely in English. A few non-English articles, such as those in Portuguese, Spanish, and French with English abstracts, were captured in the search but excluded during the full-text screening stage.

#### Search sources

We searched citations in six peer-reviewed databases: Web of Science Core Collection (list of. indexes are provided in Additional file 2), Scopus, Directory of Open Access Journals, Science Publishing Groups, AGRICOLA National Agricultural Library and Citation Database, and AGRIS Agricultural Science and Technology Information Systems. To capture articles not published in peer-reviewed sources, we also searched 22 gray literature sources. Where applicable, we set limitations on the language of the article, publication period, and type of articles during the search. Systematic map 1 and map 2 shared similar peer-reviewed databases and gray literature sources. However, each map had its search strings, thus the search process was performed separately. We listed search sources and documented the search processes for systematic map 1 and map 2 in Additional File 2.

#### Search results

Citations from each database were downloaded in the RIS file format and consolidated in the Mendeley desktop library. Mendeley aided in ensuring the completeness of bibliographic information, including authors, titles, abstracts, keywords, and journal names, through ‘update details’ feature. Citations with incomplete bibliographic information were not imported into Colandr. Regarding duplicate citations, we used Microsoft Excel to detect and eliminate duplicates before importing them into Colandr, a machine-learning tool.

### Article screening and study eligibility criteria

#### Screening process

Citations were screened separately according to the different sets of eligibility criteria of respective Map 1 and Map 2. Two different groups were assigned to perform screening stages at all levels for systematic map 1 and map 2. Furthermore, as described in the protocol paper, the screening process at the title, abstract, and full-text levels of this study was assisted by Colandr. This tool ranked the citations based on their relevance against the inclusion criteria, enhancing screening efficiency [28]. Each citation with complete bibliographic information was imported into Colandr and screened by the reviewer team. Given the number of citations collected for Maps 1 and 2 (11,538 in total), independent dual screening at the title and abstract level was done as much as 6.9% of the total 11,538 citations. The rest of the citations were screened by one person only. Any discrepancies in the screening outcomes were discussed before proceeding to the subsequent screening phase.

Subsequently, titles and abstracts that met the eligibility criteria underwent full-text screening. Before this second screening stage, we downloaded the full-text articles and imported them into Colandr. To maintain reliability and comply with the CEE standard, all individual articles in Map 1 (*n* = 964) and Map 2 (*n* = 131) were independently screened by two reviewers. Discrepancies in decision results between reviewers were discussed to reach a consensus.

#### Eligibility criteria

The eligible exposure and outcome types describing the relationship pathways between agriculture and biodiversity were derived from the TABF. The eligibility criteria for Map 1 (the impacts of tropical agriculture on biodiversity) and Map 2 (the effects of biodiversity on tropical agriculture) are detailed in Tables 1 and 2, respectively.

**Table 1:** Eligibility criteria for Map 1

Eligible population Tropical agriculture in terrestrial areas producing rice, soybeans, maize, sugar cane, wheat, oil palm, oil palm fruit, cassava, bananas, seed cotton, vegetables, mangoes, mangosteens, guavas, potatoes, cotton lint, tomatoes, oranges, coffee, yams, rubber, beans, onions, plantains, chilies and peppers, okra, groundnuts, pineapples, chickpeas, papaya, avocado, lychees, durian, rambutan, passionfruit, coconuts, grapes, cacao, clove, and tobacco
Eligible exposure Tropical agriculture production base (i.e., environmental factors, and genetic resources) and agroecosystem management (i.e., land conversion, socioeconomic factors, natural disturbances, management of soil, pest, and water, land ownership, and crop diversification)
Eligible comparator Spatial comparator in empirical or experimental studies comprises farmland with or without the agricultural interventions, and studies comparing natural or secondary forests versus farms with the interventions. Furthermore, studies comparing similar types of crop agriculture in different locations (e.g., fields, regions, or countries) will be considered. Temporal comparators examine the difference in outcomes before and after an agricultural intervention or natural disturbance exposure or compare exposures at different points in time (e.g., seasonal changes). The comparisons include different agroecosystem management methods (comparators within group of exposure) applied by farmers. For instance, comparators within groups in the exposure type of soil management are soil tillage versus chemical fertilizer amendment, in the crop diversification exposure type are polyculture with timber versus spice trees, and in the pest management exposure are pest management with natural fallows versus planted fallows, or use of different types of herbicides, and insecticide, etc.). Value ‘studies with other types of comparators’ was coded for studies that have a comparator but not the comparator within the group of exposure type we initially defined as eligibility criteria
*Eligible outcome* Changes in biodiversity indicators (e.g., species richness, abundance, composition, and density) in agroecosystems and natural ecosystems
*Eligible study types* Empirical, qualitative, quantitative, systematic reviews, reviews, experimental, quasi-experimental, and empirical modeling methods

**Table 2:** Eligibility criteria for Map 2

Eligible population Same as in Map 1
Eligible exposure Existence of biodiversity (e.g., species richness, abundance, composition, and density) and mediated functionality (i.e., ecosystem services/disservices) in agroecosystems
Eligible comparator Spatial comparator includes agroecosystems across different spatial locations. Within the group of exposures, the comparators were agroecosystems without, or with (either less or more) biodiversity mediating functionality. Temporal comparators involved comparing exposures at different points in time. Label ‘studies with other types of comparators’ were coded for studies that have a comparator but not the comparator within the group of exposure type we initially defined as eligibility criteria
*Eligible outcome* Changes in tropical agricultural production base (i.e., availability of edaphic and climatic factors and availability and suitability of crop varieties) and agroecosystem management suitability (i.e., management of soil, water, pest, and crop diversification)
*Eligible study types* Same as in Map 1

#### Study validity assessment

Critical appraisal, involving internal and external validity, was omitted. Nevertheless, we recorded information on the study setting (in situ or ex situ), study type (observational or experimental), and design comparator (existence of comparator within groups and type of comparator) from each included article [25]. Subsequently, we analyzed and presented this information in the results section (see the subsection on the quality of mapping studies relevant to the research questions).

#### Data coding strategy

Articles that passed the full-text screening stage were extracted using a previously developed coding form to obtain the necessary information. Before the coding began, a meeting for coders was held to explain how to extract articles using Colandr and to align coders’ understanding towards the coding sheet to consistently extract the required information. Additionally, a pilot test was conducted to avoid discrepancies and ambiguities in understanding the coding form and extracting the data. Four researchers participated in this test using a set of 10 articles for each of Map 1 and Map 2 that we agreed met the eligibility criteria independently of each respective Map. Any inconsistencies identified during the test were collaboratively discussed and resolved before proceeding to the data extraction stage.

Apart from the pilot test, we updated the coding form based on the feedback we obtained during the protocol paper review. The coding sheet covers several information clusters, including bibliographic information, tropical rainforest types, exposures, outcomes, agricultural commodities, study design, type of comparators, existence of comparator groups, and farm ownership. Each cluster has variable names, its definitions, and value explanations as shown in Additional file 5 in the protocol and in a ‘ReadMe’ tab in Additional file 3. Standardized and predetermined value was employed for encoding several variables such as publication types, publication content, agricultural commodity types, rainforest categories, study design, type of comparator, type of ownership, and country of which the study was conducted (i.e., using ISO standard 3166-1 alpha 3 code). For types of exposure, outcome, and existence of a comparator group, the allowed value was simply a ‘yes’ or ‘no’ option. We made each different type of exposure and outcome as an individual label in Colandr, thus coders could select more than one type of exposure and outcome based on the eligible information they extracted from the articles.

For data records, besides the predetermined value options, we put additional options of ‘other’ in the variable of rainforest type, farm ownership, and comparator group. This was due to the details provided in the articles sometimes not explicitly writing the questioned information and the description provided was not the same as what was written in the code form. For example, in the case data sampling was conducted in tropical rainforest in Malaysia but it did not mention rainforest type or describe the study site in detail such as using altitude range or physiognomic properties, coders opted ‘other’ option to avoid uncertainty in coding the right information.

Data extraction was performed in Colandr, with the aggregated data subsequently exported into a CSV file for accessibility by all research team members. Given the large volume of articles requiring extraction, each article was extracted by a single reviewer. We also did not perform formal statistics on the repeatability of data coding. However, to check the completeness of extracted information, and ensure that all coders had the same understanding of coding the information, approximately 80% of the extracted articles of Map 1 and Map 2 underwent rapid crosschecks by one reviewer at the end.

Additionally, in searching for missing or unavailable data, we did not contact the authors of the article to obtain any information that could not be found within the text or in the supplementary materials. In that case, missing information was coded as an ‘other’ value.

#### Data mapping method

The extracted data were stored and processed using an Excel spreadsheet (Additional File 3). The intersection of exposures and outcomes is displayed in heatmap 1 and heatmap 2. Heatmap 1 gathers the evidence of the impact of agriculture on biodiversity, and heatmap 2 collects the evidence of the impact of biodiversity in agriculture, respectively. These two heatmaps together are evidence of two independent unidirectional relationships. Exposures of tropical agricultural production were placed as columns, and the outcomes of biodiversity were placed as rows in the heatmap 1, and vice versa for heatmap 2. The color of the cells indicated the frequency of examination, with darker colors suggesting knowledge clusters and lighter colors indicating knowledge gaps. Notably, since one article may examine more than one type of exposure and outcome, a single article was mapped into more than one type of relational cell.

## Review findings

### Descriptive statistics review of Map 1 and Map 2

#### Number of articles

**Fig. 2:**
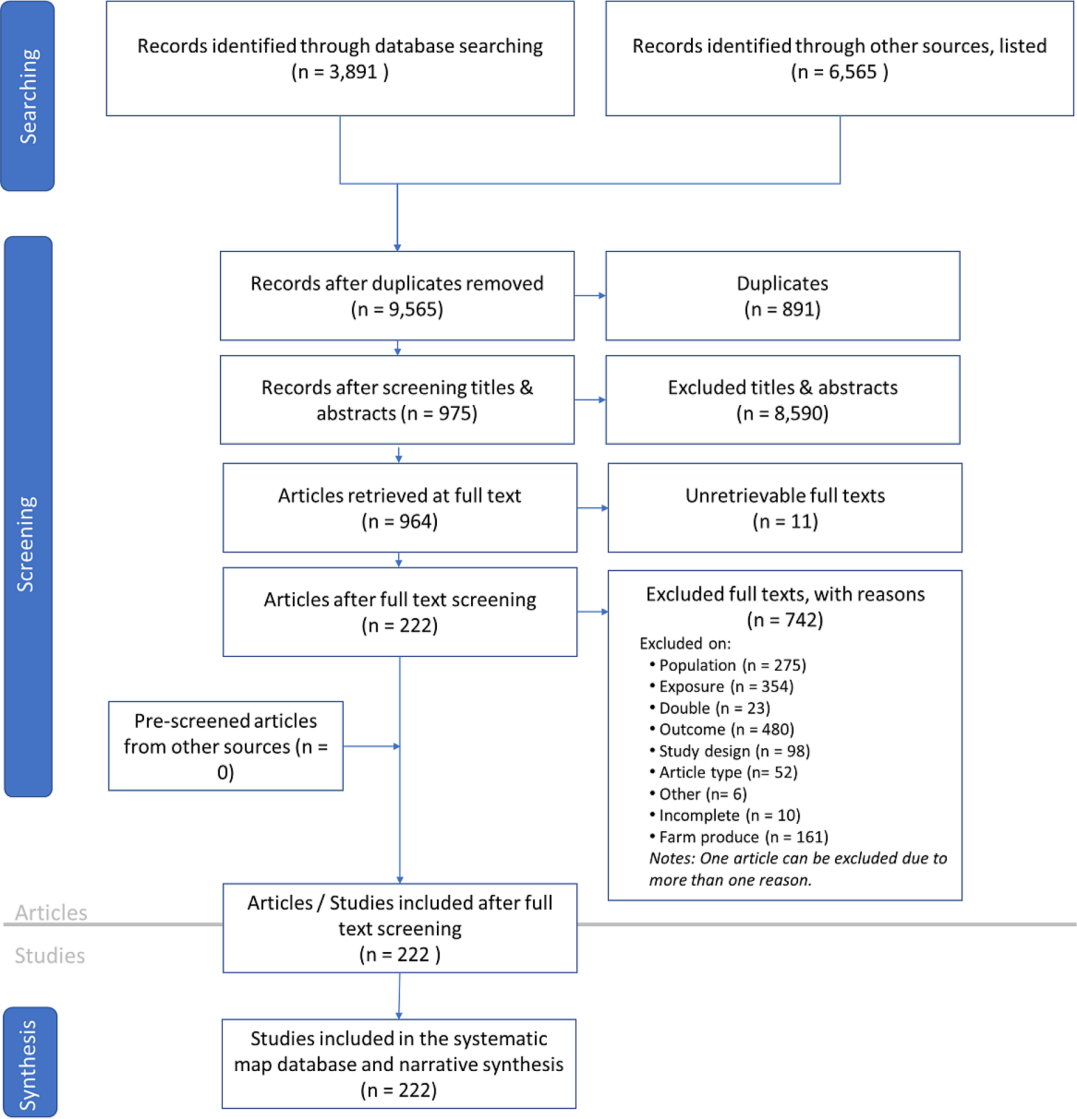
ROSES flow diagram for Systematic Map 1 (the impacts of tropical agriculture on biodiversity) illustrating the number of citations obtained from database searching, which were subsequently screened at the title, abstract, and full-text levels and extracted in the data coding stage

The systematic mapping in Map 1, focusing on the impacts of agriculture on biodiversity, is presented in Fig. 2. The search, conducted in peer-reviewed and gray literature sources during January, February, and August 2021, resulted in 3,891 citations from the peer-reviewed database and 6,565 studies from gray literature sources. A total of 8,590 retrieved citations were excluded during the title and abstract screening stages, yielding 975 eligible citations. Among these, accessible full-text articles were downloaded for the second screening stage.

Full-text articles were then screened against the inclusion criteria, resulting in 753 studies being excluded. One article can be excluded due to more than one reason. The top three reasons for exclusion were related to outcome, exposure, and population criteria. After removing ineligible studies, 222 articles were included in the data extraction stage. Prescreened articles from database sources not initially registered in the protocol paper were excluded.

**Fig. 3:**
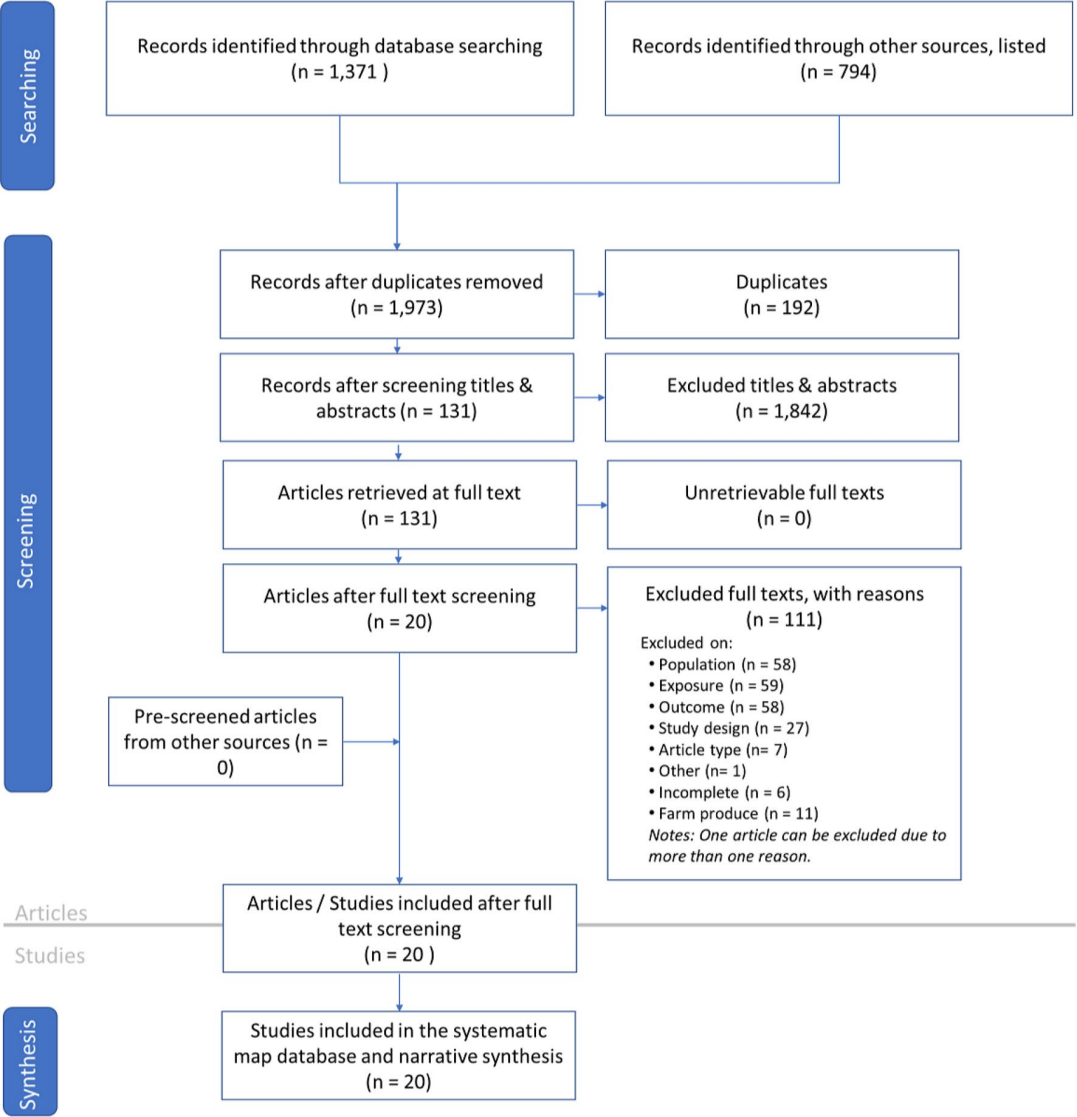
ROSES flow diagram for Systematic map 2 (impacts of biodiversity on tropical agriculture production) illustrating the number of citations obtained from database searching, which were subsequently screened at the title, abstract, and full-text levels, and extracted in the data coding stage

The systematic mapping of Map 2, which explores the influences of biodiversity on tropical agriculture management, is presented in Fig. 3. The search in peer-reviewed and gray literature sources conducted in January, February, and August 2021 resulted in 2,165 references (1,371 from peer-reviewed references and 794 from gray literature articles). Out of the total peer-reviewed and gray literature references, 1,842 references were excluded during the title and abstract screening, leaving 131 references to be screened at the full-text level.

Similar to Map 1, full-text articles were screened against the inclusion criteria, resulting in the exclusion of 111 studies, primarily due to issues related to outcome, exposure, and population type. We extracted data from 20 articles for the synthesis stage. The list of articles included in Map 1 and Map 2 is presented in Additional File 4, while articles excluded at the full-text screening, along with the reasons for exclusion, are shown in Additional File 5.

### Mapping the quantity of studies relevant to the question

#### Distribution of articles based on publication year

**Fig. 4:**
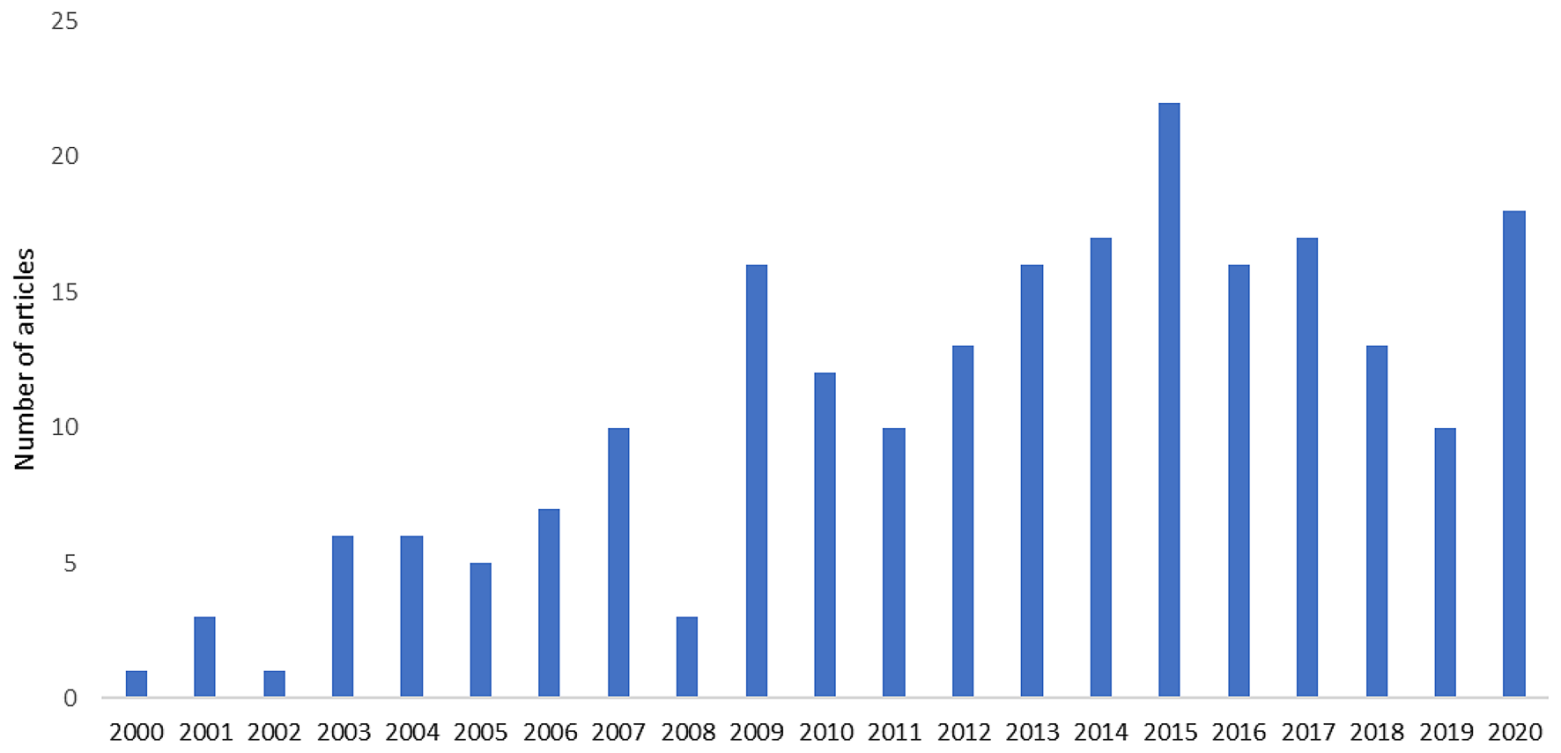
Distribution of articles in Map 1 based on the year the studies were published (2000–2020)

Trend of publication year in the last two decades were observed. In the first decade (2000–2010), the number of articles published fluctuated with the overall trend of increment. Year of 2009 had the highest number of published studies. In the second decade (2011–2020), the publication trend increased from 2011 to 2015, followed by a decline in 2016 until 2019 before it reached a resurgence in 2020 of which 18 articles were published (Fig. 4).

**Fig. 5:**
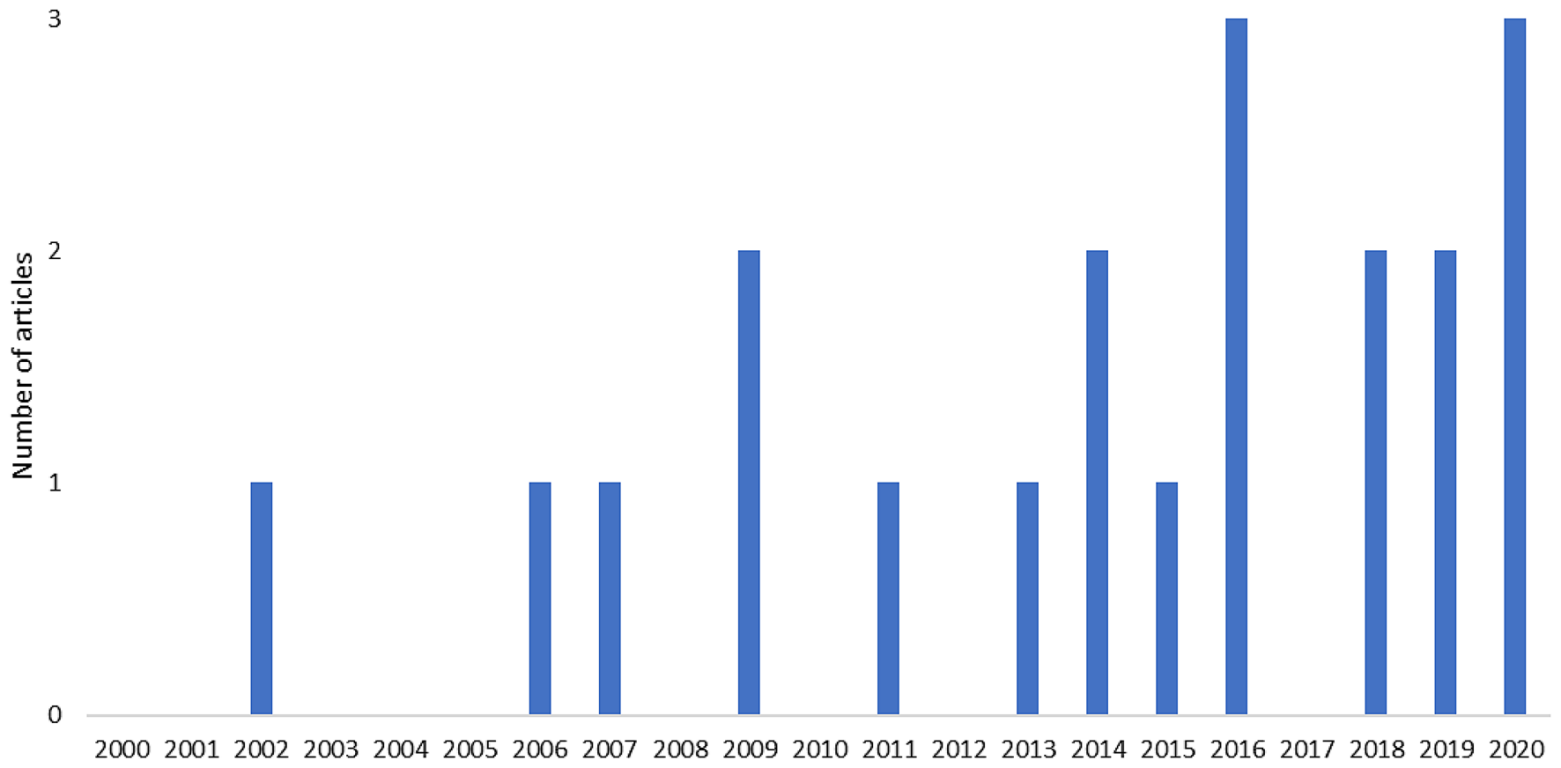
Distribution of articles in Map 2 based on the year the studies were published (2000-2020)

Similar to the trend in Map 1, in general, we observed an increasing trend in the number of articles published in Map 2. In the first decade, between 2000 and 2010, on average, only one article was published per year. In the second decade, the number of published articles showed a growing trend. Although no articles were published in 2010, 2012, and 2017, the average number of articles published in 2016, 2018, and 2020 reached two to three articles per year. However, during 2000–2020 the difference between the highest and lowest number of articles published per year in absolute numbers is small (a maximum of three articles). Figure 5 Distribution of articles in Map 2 based on the year the studies were published (2000–2020).

#### Geographic maps showing the distribution of studies by country

**Fig. 6:**
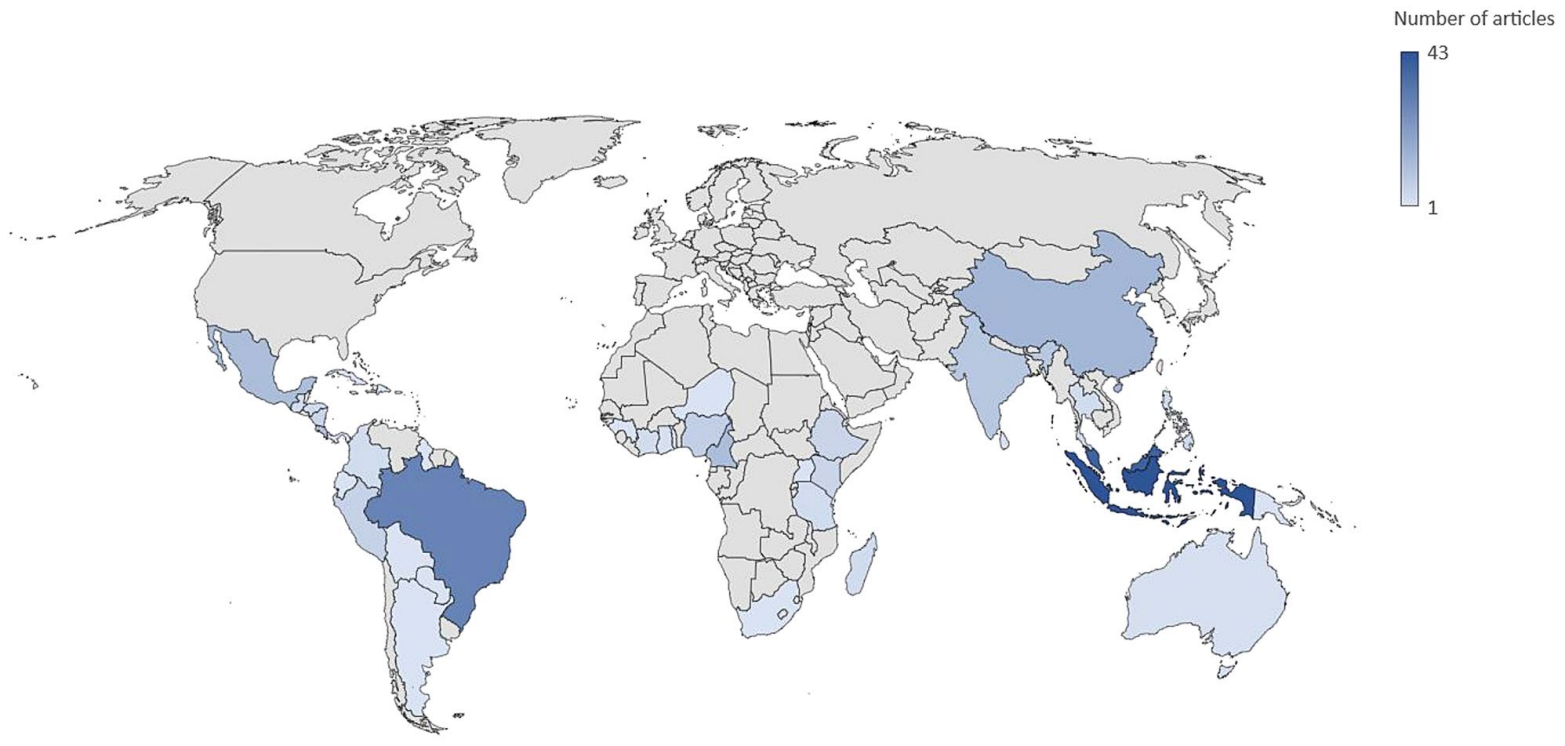
Geographic maps showing the distribution of countries examined in Map 1

Studies were conducted in all three tropical regions: tropical Latin America, Africa, and Asia. In Map 1, Indonesia, Malaysia, and Brazil were the top three countries with the most studies on the relationships between tropical agriculture and biodiversity (Fig. 6). Forty-three articles had Indonesia as their study site, while 39 studies were conducted in the neighboring country, Malaysia. Brazil was one of the countries in tropical South America with the most frequently studied sites (*n* = 29), while Cameroon was the only country in Africa that was a research hotspot (*n* = 12). This finding was unexpected, as we expected that more study sites would be found in African tropical countries, considering their significance in producing several of the world’s important commodities, such as coffee, cacao, and cotton.

**Fig. 7:**
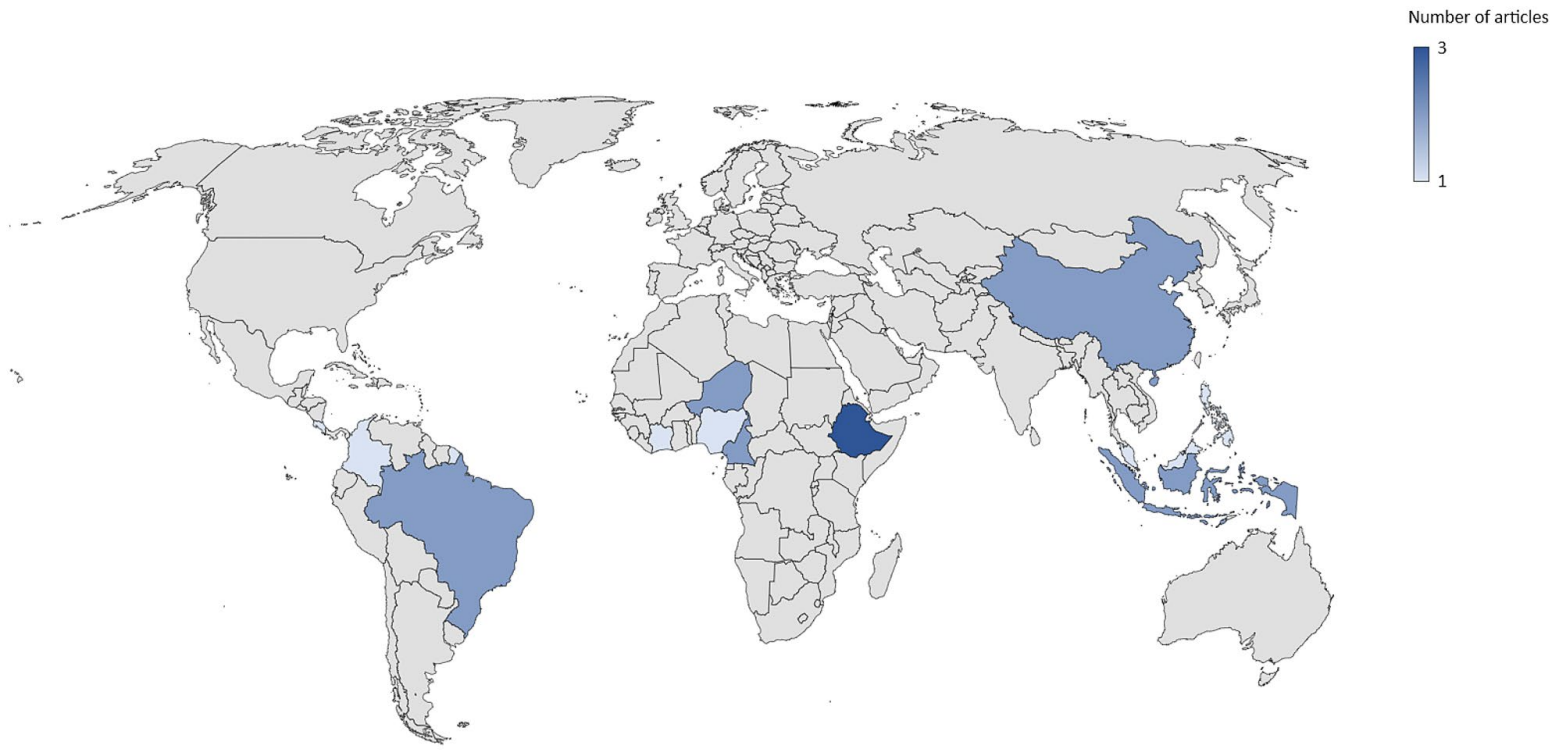
Geographic maps showing the distribution of countries examined in Map 2

Meanwhile in Map 2, the number of studies ranged from one to three, with little difference between the countries with the most and least studies. Ethiopia had the highest number of published studies, with three articles (Fig. 7).This finding was different from Map 1 where Indonesia, Malaysia, and Brazil stood as the top three countries with the most studies. In Map 2, these countries were only found in one to two articles.

#### Distribution of articles based on tropical rainforest type

**Fig. 8:**
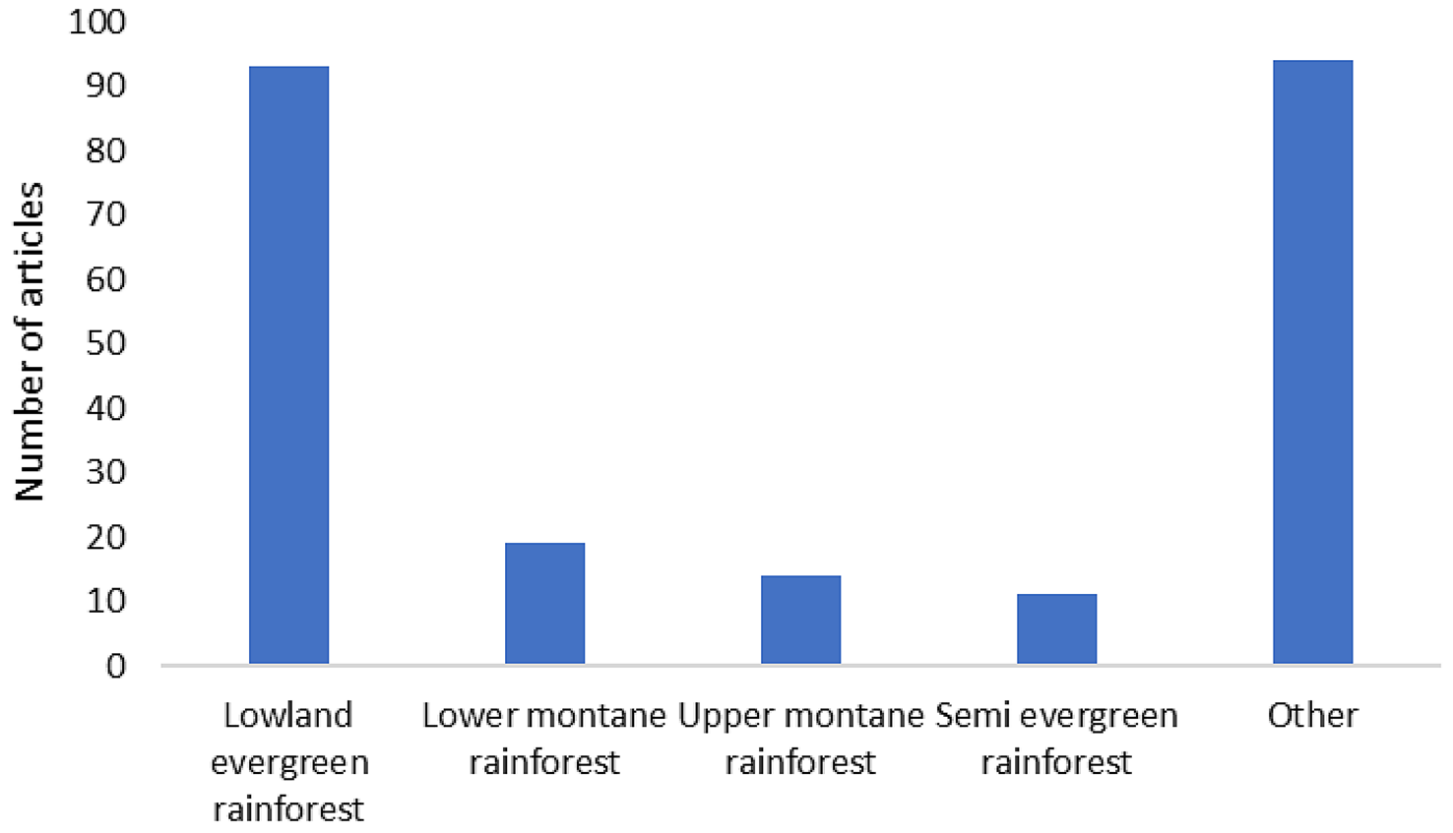
Distribution of articles in Map 1 based on the tropical rainforest type examined

Based on the rainforest categories (Fig. 8), the lowland evergreen rainforest was the most common rainforest type investigated in the collected studies (*n* = 93), followed by the lower montane rainforest (*n* = 19) as the second most investigated in Map 1. Indonesia and Malaysia emerged as the top two countries in which this rainforest type was studied. Notably, 94 articles (*n* = 94) did not explicitly mention the rainforest types listed in the protocol paper. Therefore, they were classified under the “Other” category. Noteworthy, selecting more than one type of rainforest category in the Colandr data extraction system was allowed since one article may mention more than one type of rainforest category.

**Fig. 9:**
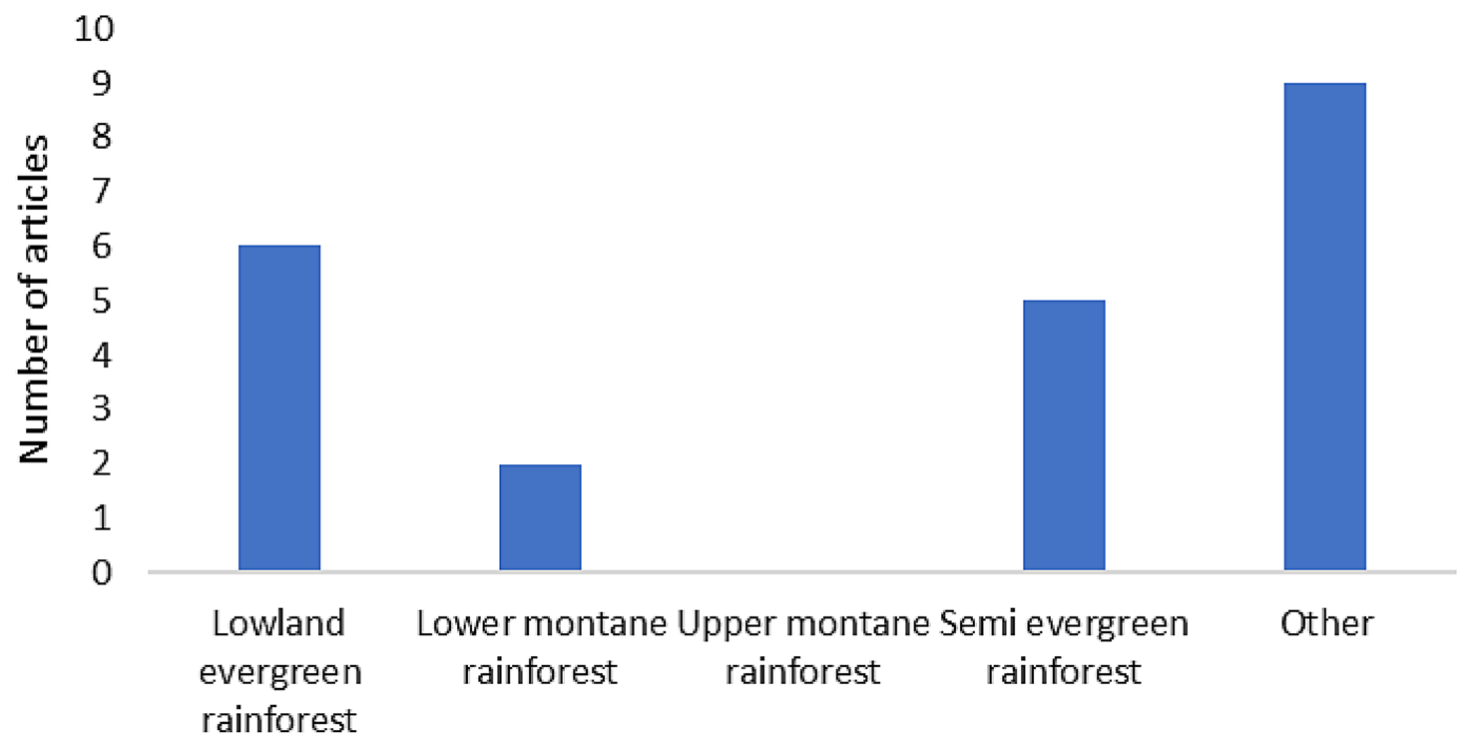
Distribution of articles in Map 2 based on the tropical rainforest type examined

Slightly different to Map 1, the lowland evergreen rainforest (*n* = 6) and semi evergreen rainforest (*n* = 5) were the first and second most common rainforest types investigated in the studies, respectively. Most of the lowland evergreen rainforests examined in the studies were in Indonesia and Nigeria. However, nine extracted articles (*n* = 9) provided no information or did not give detailed characteristics of rainforest types; therefore, we classified their study sites into the “Other” category (Fig. 9).

#### Distribution of articles by agricultural commodity

In terms of the variety of commodity types captured in the study, in contrast with Map 1 which had 30 types of farm commodities, in Map 2, only 10 commodities were identified in the articles. However, in both maps, maize, rubber, and cacao rank among the top five most studied commodities.

**Fig. 10:**
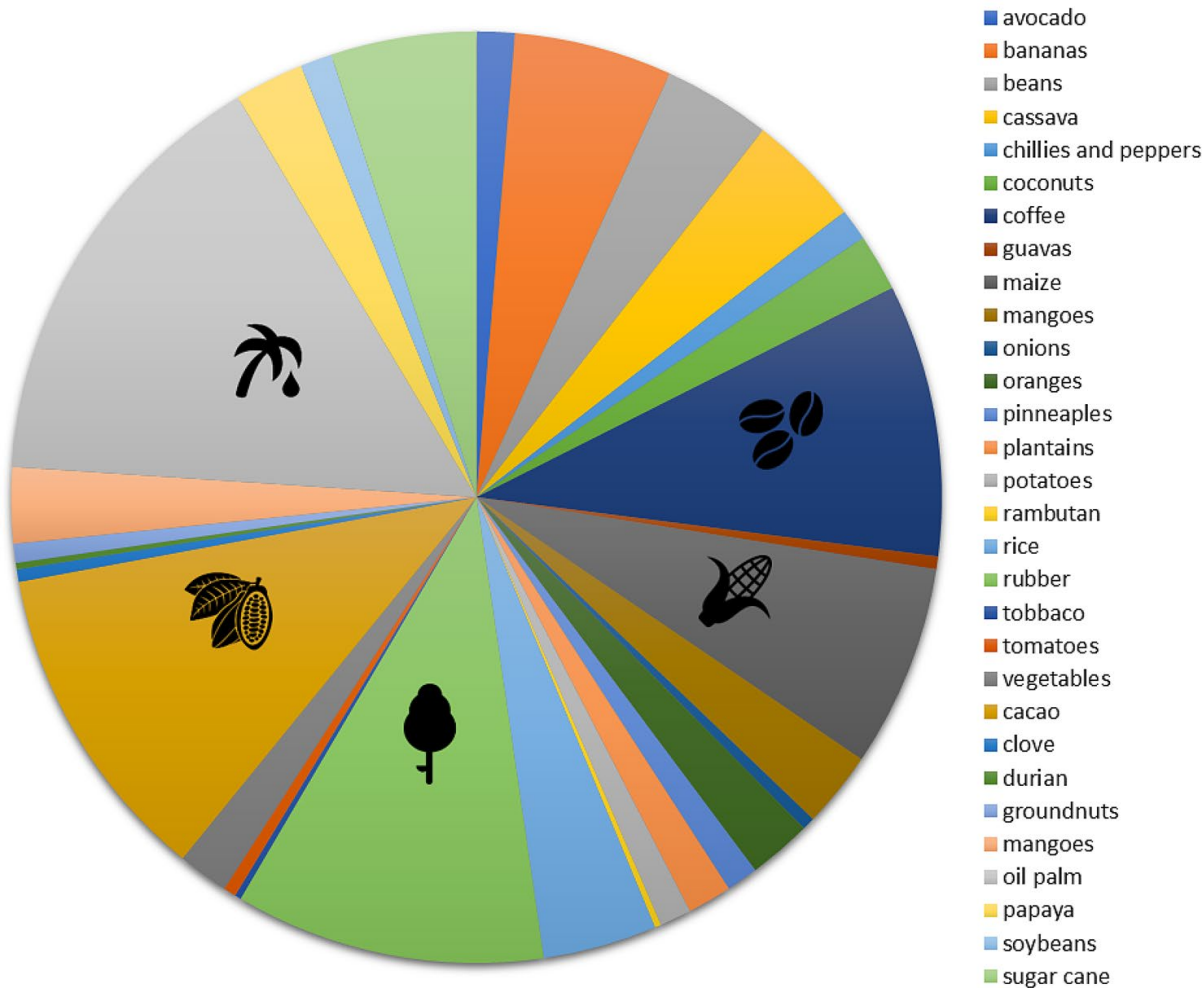
Distribution of articles in Map 1 by agricultural commodity

In Map 1, oil palm (*n* = 70), cacao (*n* = 51), rubber (*n* = 49), coffee (*n* = 43), and maize (*n* = 32) were predominantly examined in the studies (Fig. 10). These top five agricultural commodities are classified as high-economic-value commodities produced by the tropical rainforest countries recorded in this study. Specifically, Indonesia and Malaysia are the largest global producers of oil palm, while Indonesia and Thailand contribute significantly to natural rubber production. Cacao and coffee are mainly supplied by Côte d’Ivoire and Brazil, respectively. Last, China and Brazil are the leading maize producers worldwide [29].

**Fig. 11:**
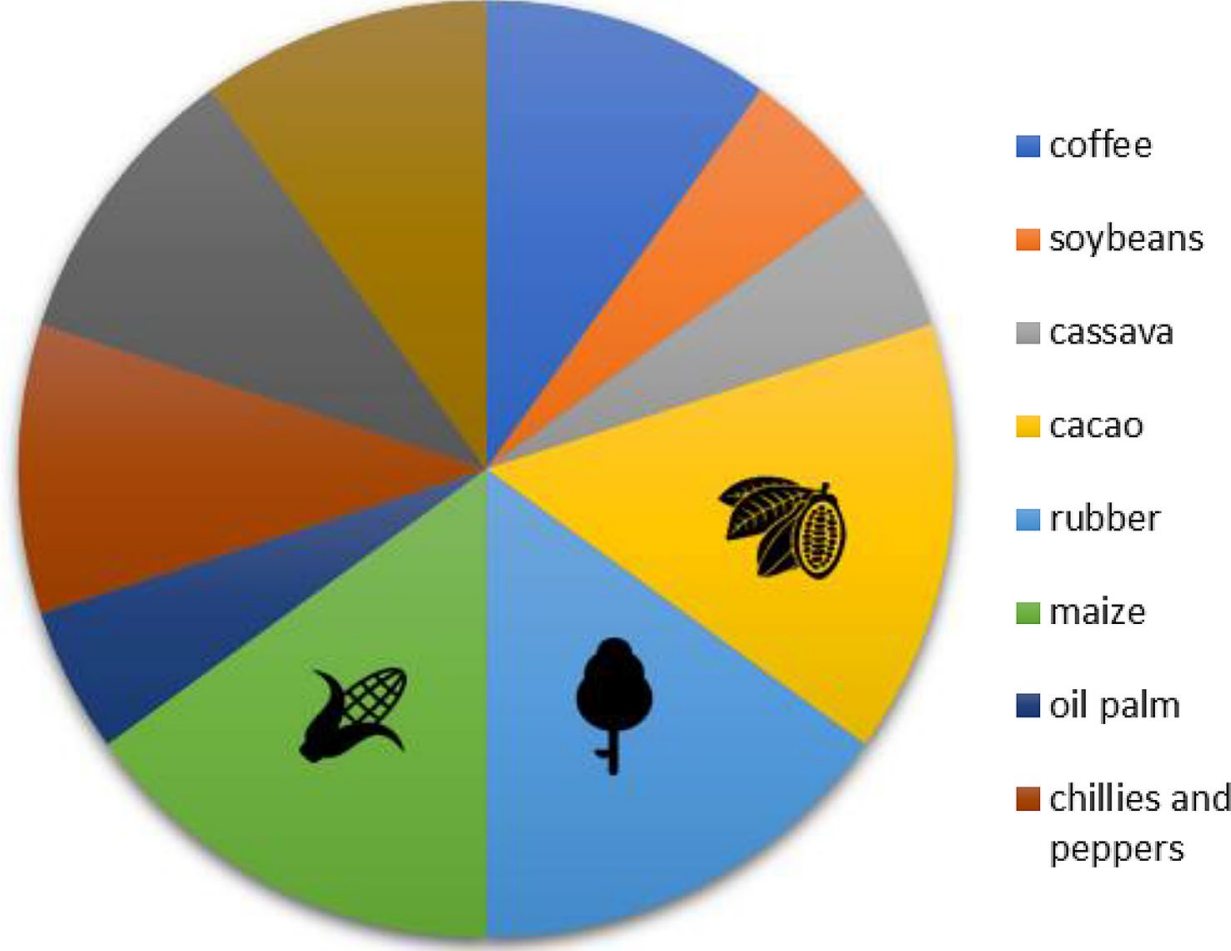
Distribution of articles in Map 2 by agricultural commodity

In Map 2, maize, cacao, and rubber were the most studied commodity (three studies each) of the 20 total articles (Fig. 11). The number of studies ranged from one to three, with no substantial difference between the most studied and least studied commodities. Notably, several articles studied more than one agricultural commodity; thus, the amount of evidence in this category did not exactly reflect the number of articles extracted.

#### Distribution and frequency of agricultural exposures examined in Map 1

**Fig. 12:**
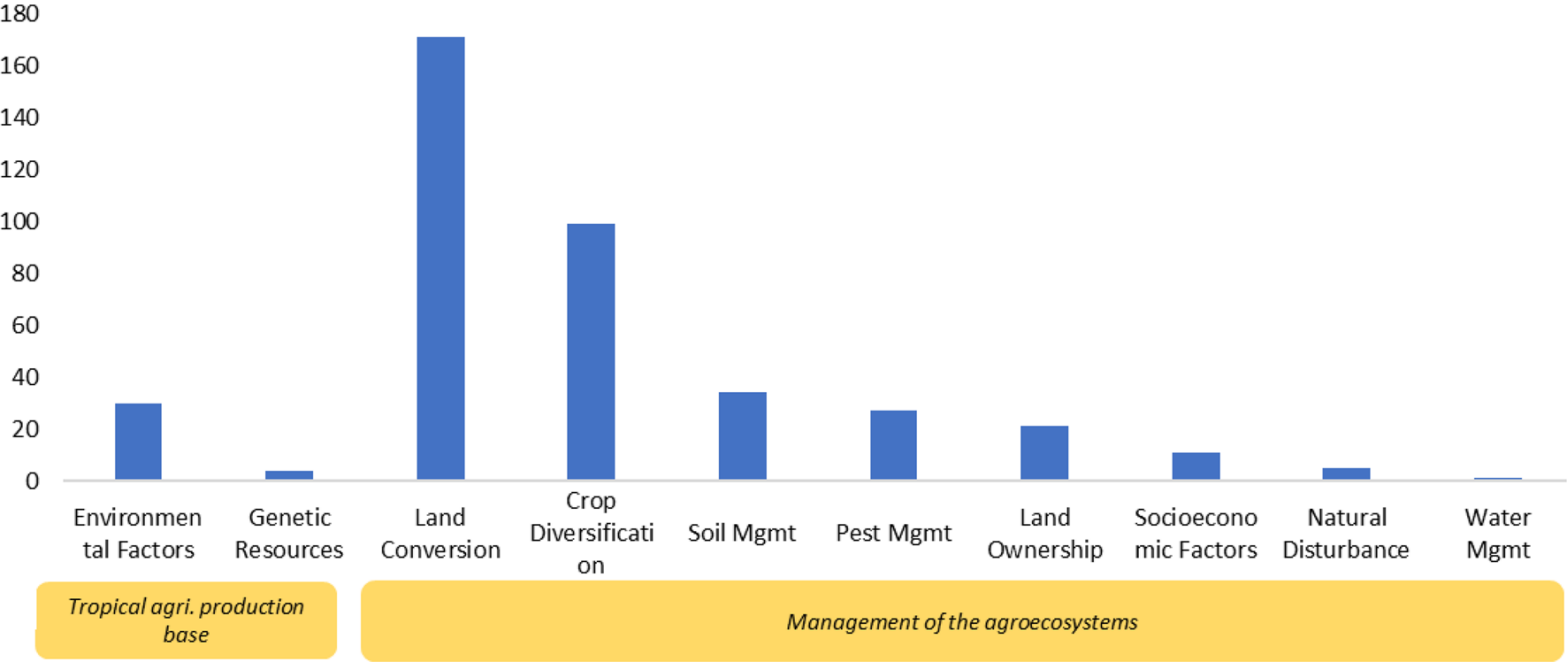
Distribution and frequency of tropical agricultural exposures examined in Map 1

Regarding exposure, land conversion and crop diversification were the most frequently studied types of agricultural exposure in Map 1 (Fig. 12). Overall, 171 studies were identified as having investigated the impacts of land conversion on biodiversity, ranking land conversion exposure as first among all exposure types. Notably, crop diversification was the second most frequently studied exposure type. We found that 99 articles studied crop diversification systems (in the form of monoculture or polyculture, such as agroforestry or crop rotation) and measured their impacts on biodiversity. The other most frequently studied exposure types included soil management, environmental factors, pest management, and land ownership. Conversely, the least frequently studied exposure types were genetic resources, natural disturbances, and water management.

#### Distribution and frequency of biodiversity exposures examined in Map 2

**Fig. 13:**
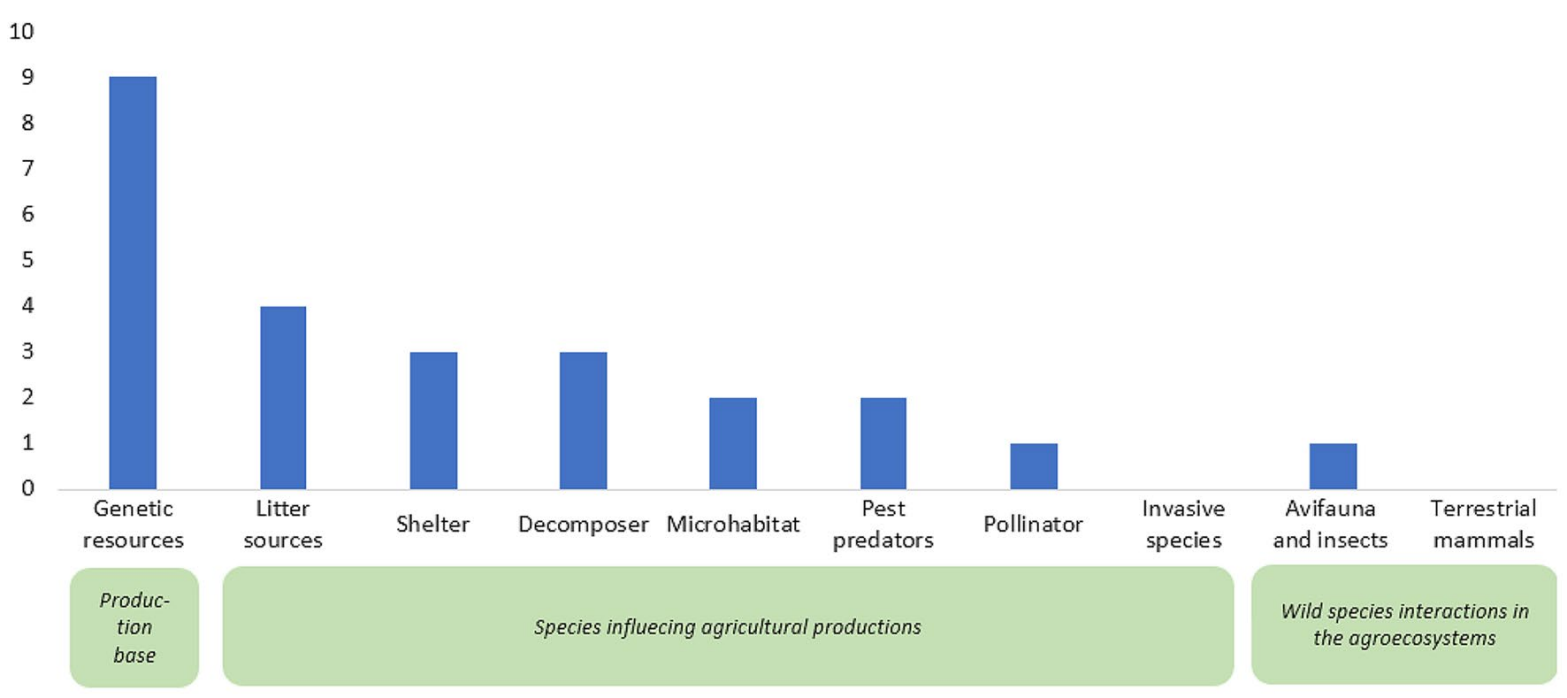
Distribution and frequency of biodiversity exposures examined in Map 2

The most predominantly studied exposure type was biodiversity as a genetic resource (*n* = 9), followed by litter sources (*n* = 4), decomposers (*n* = 3), and shelters (*n* = 3). “Genetic resources,” as an exposure type, mainly involved crop genetic varieties. Conversely, no study has focused on terrestrial mammals and invasive species as exposures (Fig. 13).

#### Distribution and frequency of biodiversity outcomes examined in Map 1

**Fig. 14:**
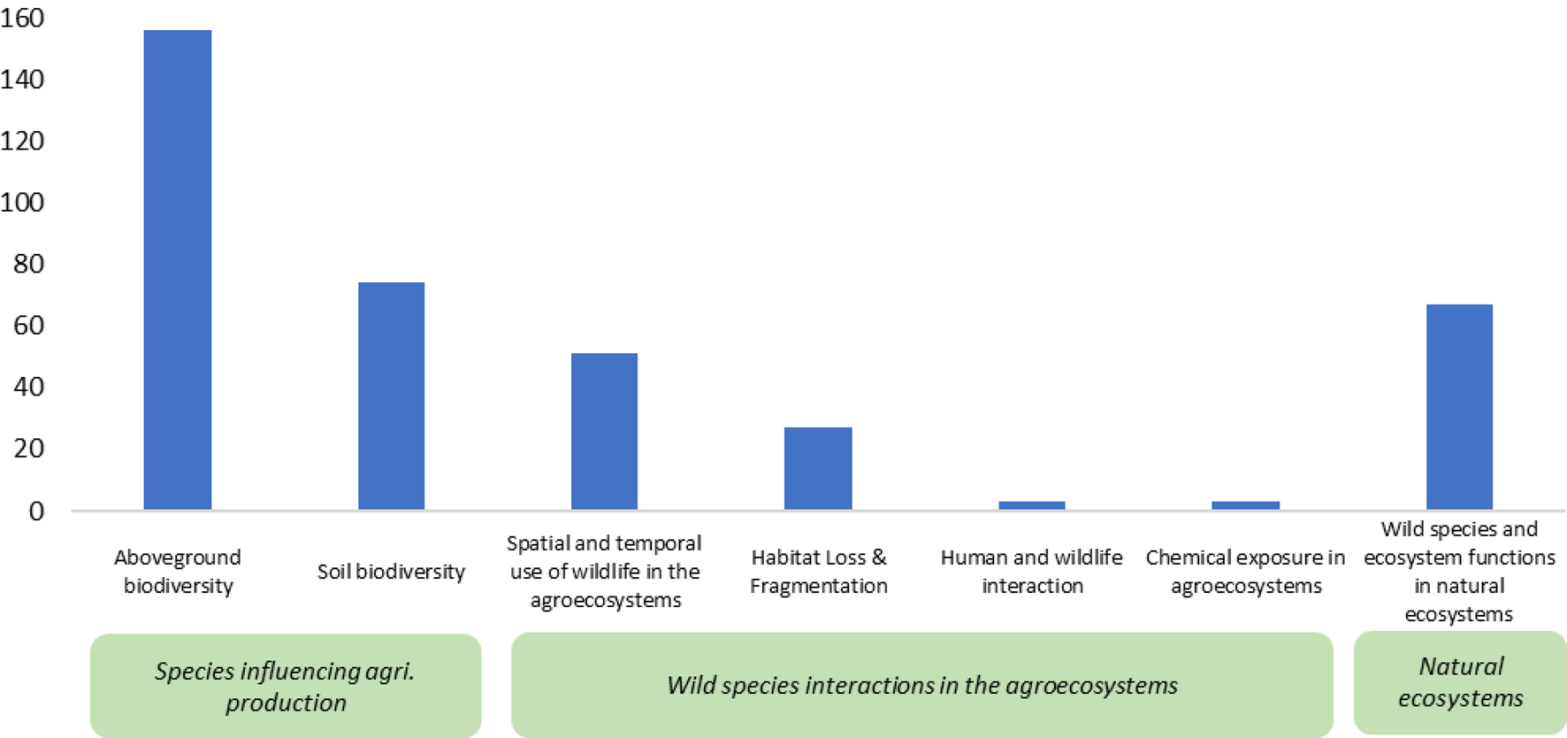
Distribution and frequency of biodiversity outcomes examined in Map 1

The top three frequently measured biodiversity outcomes were aboveground biodiversity (*n* = 156), soil biodiversity (*n* = 74), wild species and ecosystem functions in natural ecosystems (*n* = 67), and spatial and temporal use of wildlife in agroecosystems (*n* = 51) (Fig. 14). Aboveground biodiversity encompassed plants, animals, and macrofungi. Soil biodiversity comprised microfungi, microorganisms, and soil invertebrates, contributing to ecosystem services and disservices in agroecosystems. However, few studies have investigated the impacts of tropical agriculture on biodiversity through measured outcomes, such as habitat loss and fragmentation (*n* = 27), human and wildlife interaction (*n* = 3), and chemical exposure in agroecosystems (*n* = 3).

#### Distribution and frequency of tropical agriculture outcomes examined in Map 2

**Fig. 15:**
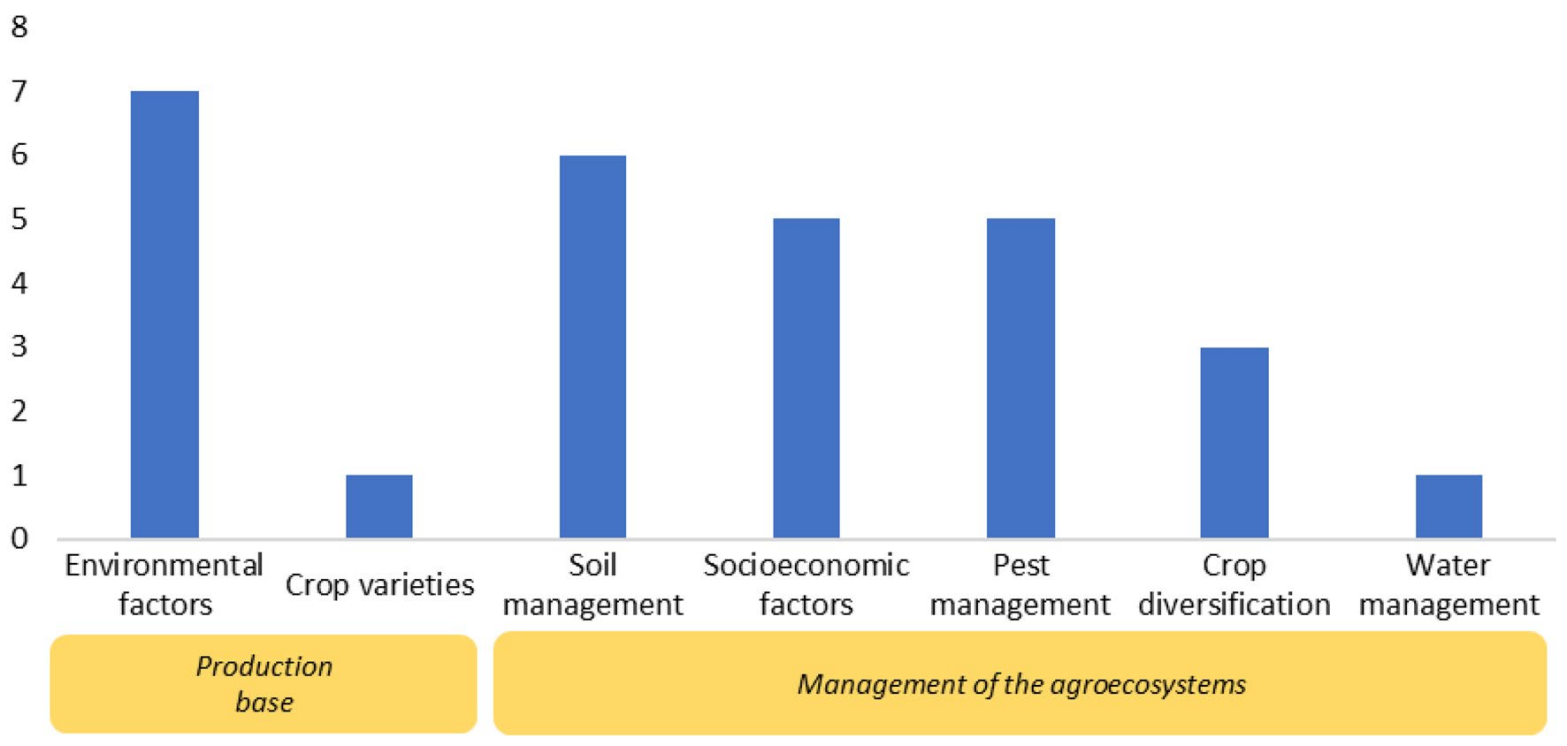
Distribution and frequency of tropical agriculture outcomes examined in Map 2

The most frequently measured outcomes were environmental factors (*n* = 7) and soil management (*n* = 6). Environmental factors in the extracted articles mostly involved the soil’s environmental condition. Moreover, only one study has investigated the impacts of biodiversity on water management and crop varieties in tropical agriculture (*n* = 1) (Fig. 15).

### Mapping the design of studies relevant to the question

#### Study design and comparator types used in the extracted articles

**Fig. 16:**
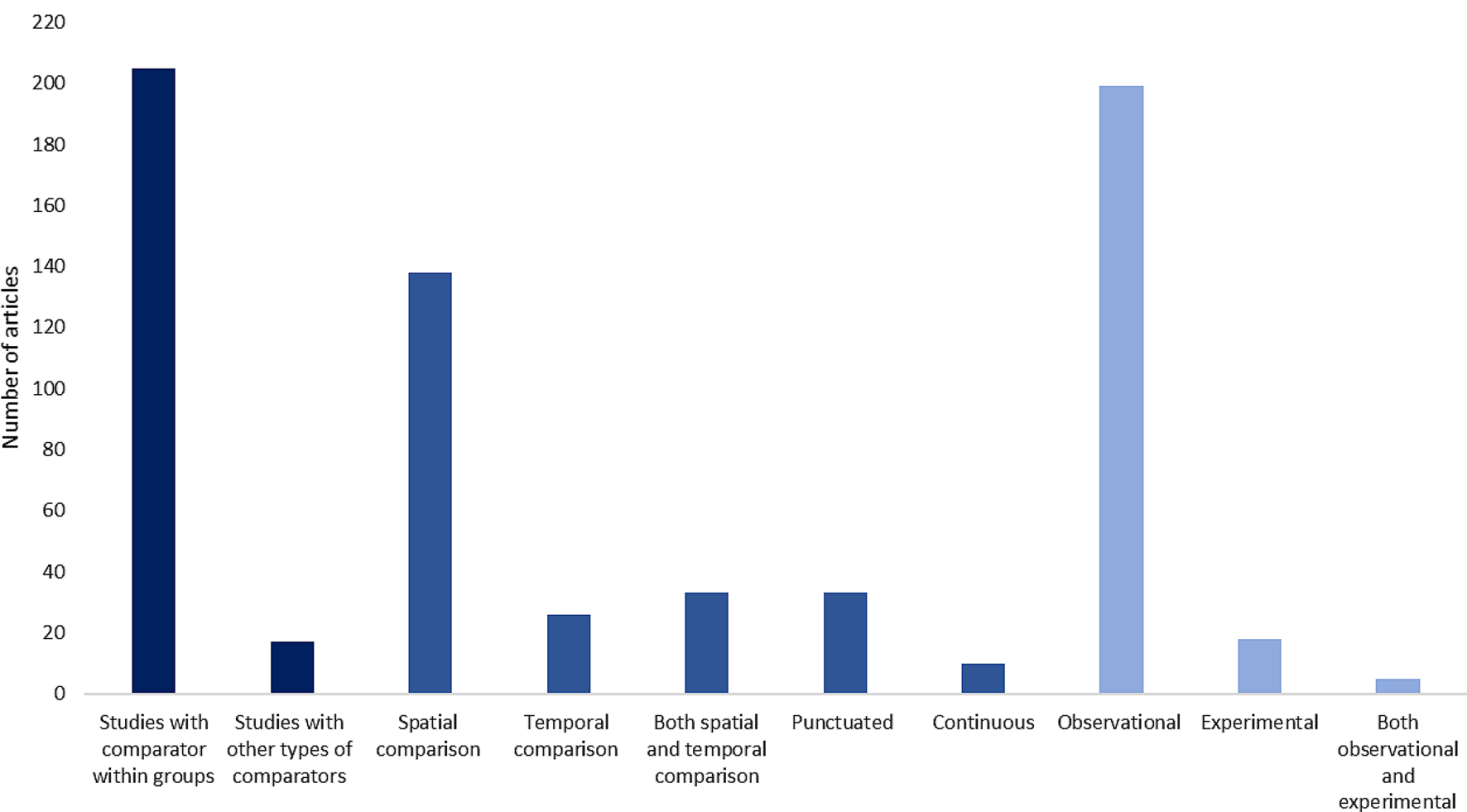
Frequency of comparator types (dark blue and medium blue colors) and study design (light blue colors) employed in the articles extracted in Map 1

To report the quality of evidence collected in this systematic map, we extracted information regarding comparator types and study design used in each included article. In Map 1 (Fig. 16), most of the evidence involved comparators within groups (92%), while the evidence with other types of comparators was 7.6%. Studies that used spatial comparison were higher (62%) than those that used temporal comparison (11%). Regarding study design, articles that employed observational methods (89.6%) exceeded those that employed experimental methods (8.1%), and those that used both observational and experimental methods (2.2%).

**Fig. 17:**
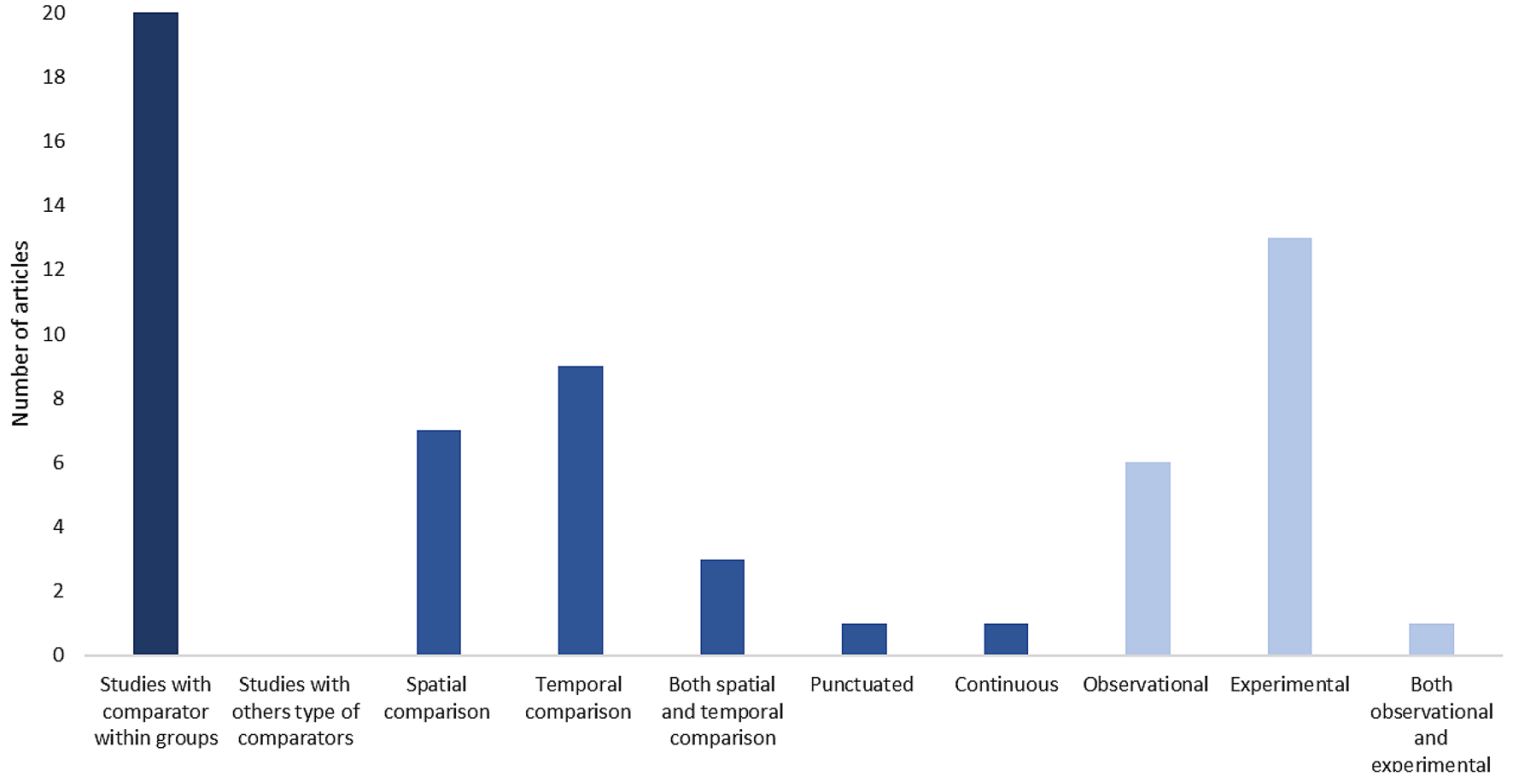
Frequency of comparator types (dark blue and medium blue colors) and study design (light blue colors) employed in the articles extracted in Map 2

The comparator types and study design recorded from Map 2 were contrary to the data recorded from Map 1. In Map 2, all the studies used a comparator within groups in their methods. The studies that used temporal comparison (45%) were slightly greater than studies that used spatial comparison (35%). Meanwhile, 15% of the evidence covered in Map 2 employed both spatial and temporal comparisons. Studies that used an experimental design (*n* = 13) were higher than those that employed an observational design (*n* = 6) and those that used both observational and experimental design (*n* = 1) (Fig. 17).

### Distribution of evidence in Map 1 and Map 2

**Fig. 18:**
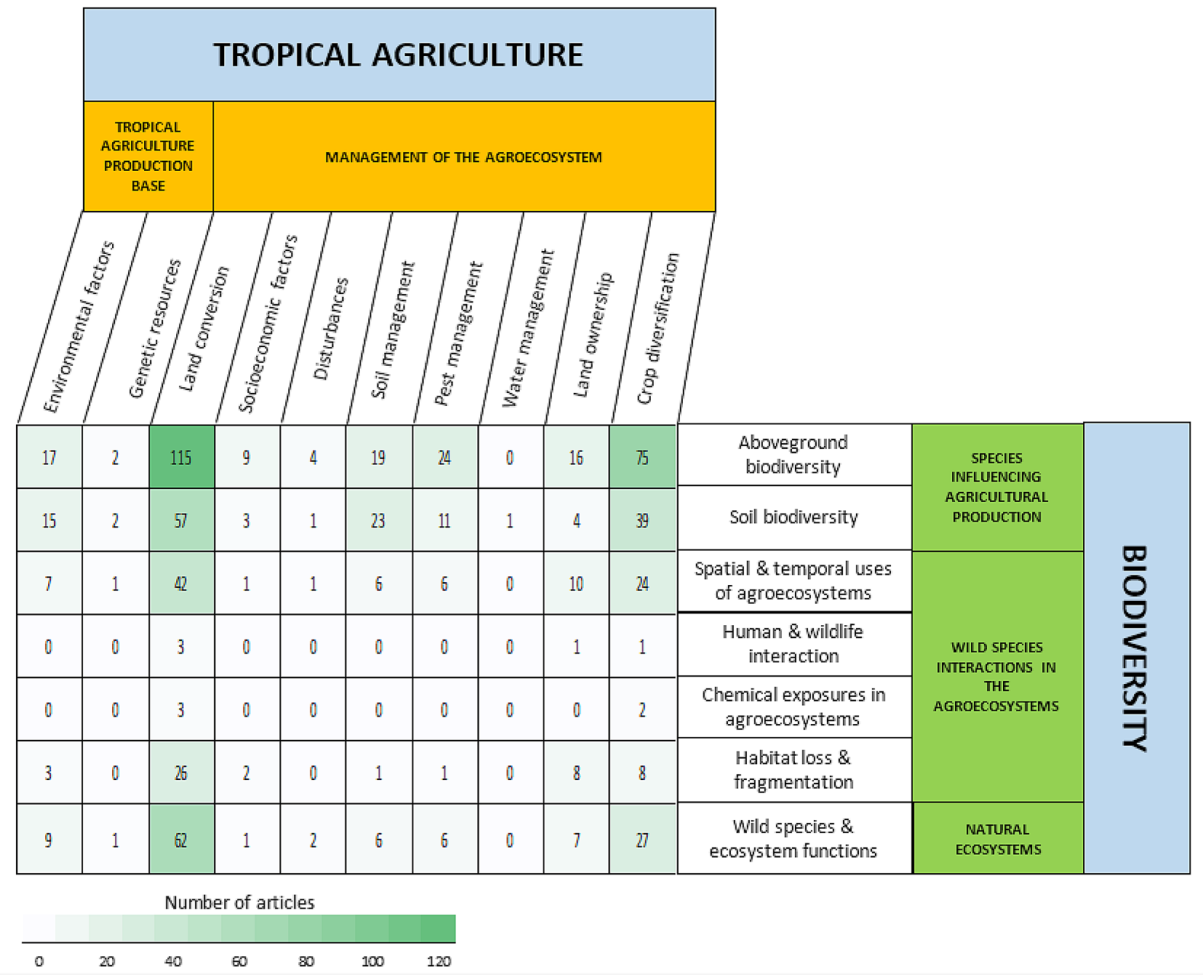
Distribution of evidence in Map 1, revealing the relationships between tropical agricultural activities and biodiversity outcomes in agroecosystems and natural ecosystems. Articles can fall into more than one linkage cell

The intersections of tropical agricultural exposures and biodiversity outcomes, indicating the relationships between both elements, are presented in Heatmap 1 (Fig. 18). The darker cells show the most examined relationships, while the lighter cells indicate significantly fewer relationships examined in the extracted articles. The impacts of land conversion (including transforming primary and secondary forests or abandoned land into cropland) on all biodiversity outcomes (except human and wildlife interaction and chemical exposure in agroecosystems) were the most commonly investigated relationship types. Specifically, 115 studies measured the impacts of land conversion on aboveground biodiversity, and 62 studies examined its impacts on wild species and ecosystem functions in natural ecosystems. The other most frequently examined linkages included the impacts of crop diversification on aboveground biodiversity (*n* = 75), soil biodiversity (*n* = 39), and wild species and ecosystem functions in natural ecosystems (*n* = 27).

Conversely, the least studied relationships included the impacts of all exposure types (tropical agricultural production base and agroecosystem management) on human and wildlife interactions and chemical exposures in agroecosystems. The relationships that lacked evidence included the effects of genetic resources, natural disturbances, and water management on all aspects of biodiversity outcomes.

**Fig. 19:**
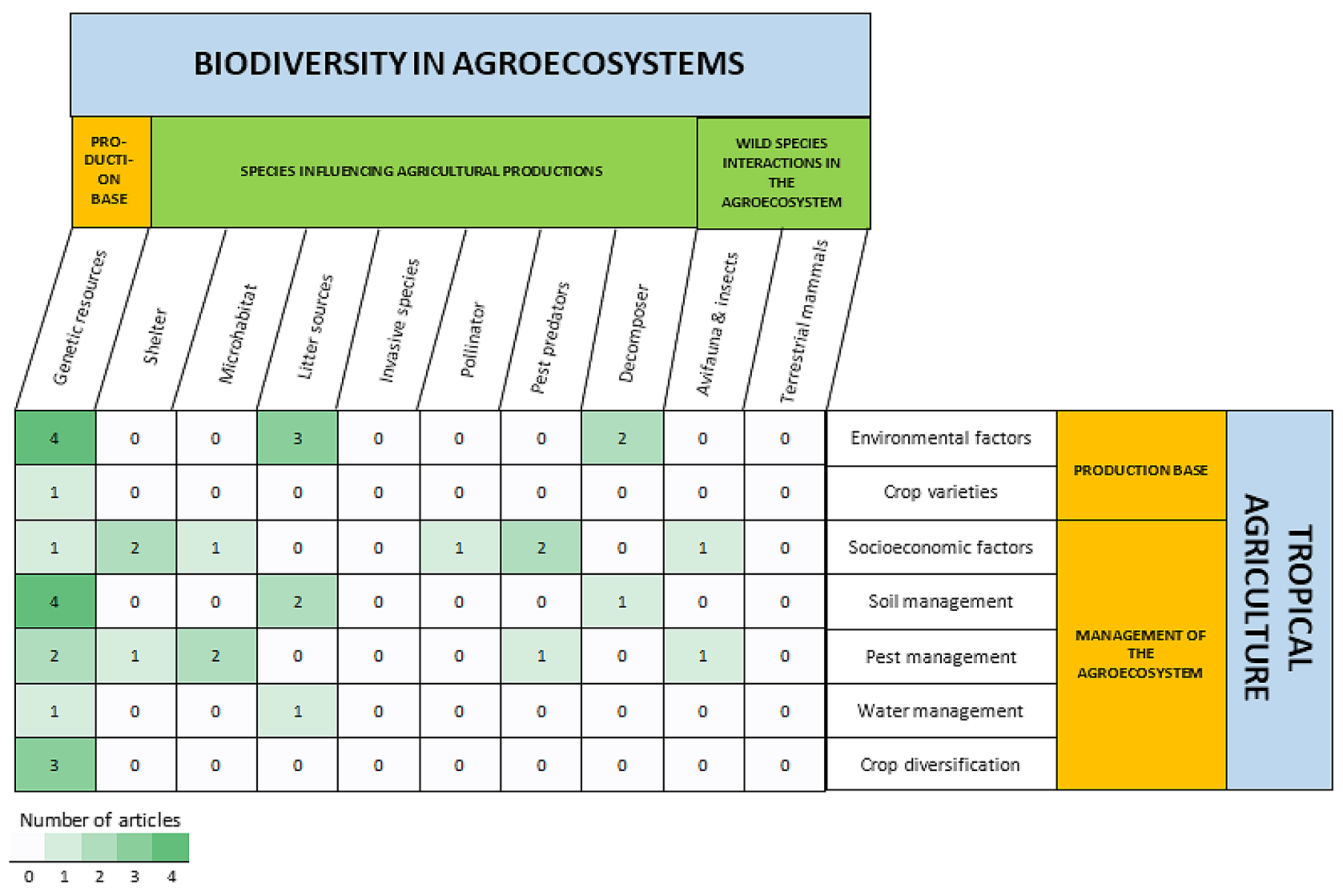
Distribution of evidence in Map 2, revealing the relationships between tropical biodiversity and the agricultural outcomes in agroecosystems. Articles can fall into more than one linkage cell

The intersections of biodiversity exposures and agricultural outcomes that indicate the relationships between both elements are presented in Heatmap 2 (Fig. 19). Most identified articles (16 references in total) focused on the relationship between crop genetic resources as an exposure type and tropical agriculture outcomes (production base and agroecosystem management), especially environmental and edaphic factors. Conversely, despite invasive species and terrestrial mammals being ubiquitous in agricultural areas, we did not find any articles discussing the impact of their presence on agricultural outcomes. Additionally, the least studied relationships were the impacts of all biodiversity exposure types on crop varieties (*n* = 1) and water management (*n* = 2) in agroecosystems.

### Knowledge clusters and knowledge gaps

Overall, compared to the amount of evidence in Map 1, the distribution of evidence examining the relationship between biodiversity and its influence, either in providing services or disservices to agricultural activities, specifically on the selected crop commodities in Map 2, has not been extensively explored. This is noteworthy, particularly for researchers interested in the tropical rainforest landscape, given the importance of ecosystem service values provided by wild species (e.g., provisioning and regulating services) to farmers [5, 30]. This is further strengthened by the need to consider biodiversity within the context of crop production, as echoed in the post-2020 biodiversity and Sustainable Development Goals (SDGs) targets.

The most conspicuous gap in Map 2 compared to Map 1 is there were only 20 articles collected and coded in Map 2. Meanwhile in Map 1, there were 222 studies. The overall lack of evidence of the impact of biodiversity on agricultural production (Map 2) in this systematic map is noticeable. In terms of types of exposures and outcomes, two potential literature gaps were identified specifically: (1) the effects of the presence of terrestrial mammals and invasive species in agroecosystems on agricultural outcomes, and (2) the relationship between the impact of biodiversity exposure on water management and crop varieties as the outcomes.

Findings in Map 2 also revealed that there were more studies examining ecosystem services provided by biodiversity than those evaluating ecosystem disservices provided by biodiversity in agriculture. Many studies have investigated the benefits of biodiversity to agriculture, as evidenced by the high distribution of evidence examining the role of biodiversity as genetic resources (i.e., drought or pest-resistant cultivars), shelters, litter sources (e.g., a variety of trees, herbs, and shrubs that affect soil nutrient), and decomposers (e.g., termites, ants, and earthworms) in agricultural production and management. Conversely, no evidence investigating the relationships between invasive species (ecosystem disservices) and agriculture.

In the systematic map of evidence in Map 1, our study results highlight two gaps as follows: (1) the relationship between the impacts of all agricultural exposure types on human and wildlife interaction and chemical exposures in agroecosystems, and (2) changes in biodiversity outcomes due to genetic resources, natural disturbances, and water management. Additionally, compared to more technical and agronomy-centric exposure types, such as soil and pest management, there is less evidence investigating the relationships between socioeconomic factors and biodiversity outcomes. Thus, we encourage researchers and scholars to further explore socioeconomic aspects and examine how much these factors influence farmers’ decisions toward crop management or agricultural practices, which could impact biodiversity conservation or decline. The primary evidence based on these research topics could be performed using a transdisciplinary approach involving experts from social, economic, agricultural, and ecological backgrounds [31, 32]. This collaborative transdisciplinary approach will allow researchers to share and integrate scientific knowledge and methods to better comprehend the complexity of issues regarding agriculture and biodiversity [5, 33].

In addition to evidence gaps, several knowledge clusters can be inferred notably from Map 1, which are the impacts of land conversion on aboveground biodiversity and wild species and ecosystem functions in natural ecosystems. The other most studied relationships were crop diversification effects on aboveground biodiversity, soil biodiversity in agroecosystems, and wild species and ecosystem functions in natural ecosystems. Following this, a more thorough synthesis or review study could be conducted to explore causative processes, assess their significance, and observe the nature of the relationship within these topics. For example, by focusing on geographic areas or types of commodities. Through such a study, more informative results can be concluded and practically be used in helping farmers, landowners, or agriculture enterprises in taking an evidence-informed decision.

### Limitations of the map

#### Limitations due to the search strategy

Although we attempted to design and execute the search strategy as comprehensively as possible to gather existing evidence relevant to the topic, several limitations that potentially affect the results of this study persist. First, regarding the eligible publication time range established in our inclusion criteria, this study focused on articles published between 2000 and 2020 (as explained in the protocol paper). Thus, we did not include evidence published before 2000.

Second, although we strived to inclusively capture and pool evidence during the search stage, the evidence synthesized in this study might have language bias since we focused solely on studies written in English. As has been investigated by Konno et al. [34], on disregarding literature in languages other than English could introduce bias to the result of ecological meta-analyses since differences in effect sizes between studies published in English and those in non-English languages may occur. Searching in English may capture articles with only statistically significant results, as these articles have a higher chance of being published in English database sources [35, 36]. Our focus on searching and including only English-written articles eased constraints on time, resources, and access to databases during the review process. However, this may have led to unrepresentative results, as we may have potentially missed evidence written in non-English languages, such as French, Portuguese, Indonesian, and Malay, which are official languages of some tropical rainforest countries. Some relevant articles might be published in local journals that are accessible only in the local language. This language bias may explain one of the reasons why Cameroon stood out as the sole research hotspot in Africa, defying our predictions (Map 1 Fig. 6). Several African countries have French and Swahili as their official language, and the other types of national languages other than these two.

The other consequence of this language bias that may attenuate the results of this study is the overinterpretation of the relationships between tropical agriculture and biodiversity with abundant evidence and, conversely, the relationships with limited evidence. Considering the language barriers, we encourage using other languages for future improvements in this systematic mapping study.

Third, there are limitations related to the search terms used in the search process. Research themes on agriculture and biodiversity spanning the natural and social sciences may contain diverse terms or synonyms. The complexity of this linguistic diversity, according to Westgate [37], can hinder the systematic research process. Although we strived to reduce this linguistic bias by involving cross-sectoral review team members in formulating search strings and using the CABI thesaurus (https://www.cabi.org/cabthesaurus) to identify synonyms and related terms, we realize that the search results may not be comprehensive, and some articles that should be captured may be missed. Different databases might also require slightly different search terms, which frequently compelled us to shorten the string combinations (“tropical agriculture” AND “biodiversity”). This occurs due to limited search functions, commonly encountered in gray literature sources and a few peer-reviewed databases, making the article searching process less consistent.

Lastly, we recognized that the way we produced the test libraries was subject to author bias. This occurred as the test libraries were created by the research team considering the expertise and deep understanding of the conceptual framework and eligibility criteria that guided this study. Although our test lists were independent of the search process, the CEE guideline encourages authors that the lists are created by asking experts, researchers, or relevant stakeholders, and through scrutinizing bibliographic list of existing systematic maps or reviews within the topic of interest [26].

#### Limitations due to bias in the evidence found

In addition to the limitations in the search strategy, we acknowledge further limitations due to inherent biases found in the evidence pooled in this study. Since we did not conduct a critical appraisal of study validity, we could not assess confounding variables that might affect the conclusions deduced from each article, potentially introducing bias to the results informed by this systematic map. However, we recorded information on the study design (observational or experimental), the research context (in situ or ex situ), the presence of a comparator within the group of exposure and outcomes, and comparisons over spatial and temporal dimensions.

We excluded articles applying solely qualitative, conceptual, and philosophical approaches since these methods lack generalizability and transferability [38]. However, this may likely reduce the amount of collected evidence measuring the impacts of socioeconomic factors on biodiversity outcomes (Map 1), as research within this subtopic commonly employed purely qualitative approaches, as demonstrated in Kekeunou [39] and Therville et al. [40].

For Map 2, we acknowledge that it was produced from a relatively small number of extracted articles and that any conclusion drawn could potentially be biased. Therefore, we report the results as retrieved from the extracted articles without making strong claims.

## Conclusions

### Implications for policy/management

Map 1 highlights abundant evidence on the following themes: (1) the impacts of land conversion on aboveground and soil biodiversity and (2) the effects of crop diversification interventions (monoculture or polyculture) on aboveground and soil biodiversity. Meanwhile, the findings in Map 2 show studies on the relationships between the following: (1) genetic resources of crop plants (exposure) on environmental factors, and soil management in agroecosystems (outcome), and (2) aboveground biodiversity as litter sources, and decomposers (exposure) to environmental factors in agriculture production base (outcome).

Our collated evidence makes the existing evidence regarding the relationships between tropical agriculture and biodiversity accessible to decision makers and practitioners. Therefore, the decision-making process is expected to be evidence-informed. However, the results of this systematic map study cannot provide further information beyond which relationships are currently the most studied and which are still under-researched. Informing and concluding whether particular actions lead to the measured consequences (i.e., which agricultural exposures harm or maintain species in agroecosystems or natural ecosystems) are outside the scope of this systematic map. To provide this information, a further planned study involving a significant test that can be validated internally and externally is required [41]. This advanced review study has potential applications, especially for crop commodities with the most abundant evidence (e.g., palm oil, cacao, rubber, coffee, and maize).

In addition, the data extractions presented in this report can serve as a foundation for policymakers and institutions involved in the formulation and evaluation of sustainable agricultural practices certification. Organizations like the Roundtable on Sustainable Palm Oil (RSPO) or the Rainforest Alliance can utilize this information to assess their existing certification criteria. The information regarding the types of agricultural commodities and their relationships with agricultural exposure and biodiversity outcomes provided in this study might help them highlight which certification criteria might require improvement or specification. This, in turn, can contribute to achieving more accurate goals grounded in the existing empirical evidence collected in this study.

### Implications for potential primary and synthesis research

The pressures on tropical rainforests in Southeast Asia, Latin America, and Africa due to deforestation driven by agricultural activities [42] in past decades are predicted to continue [12]. Until at least 2050, approximately 1 billion ha of land will be required for food production [43]. These conditions threaten the natural habitats of forest species, rendering some endemic species more vulnerable to extinction [44]. Thus, assessing and monitoring tropical biodiversity conditions, especially in biodiversity hotspots where agricultural activities occur, is important. Moreover, with the SDG target 15 and the post-2020 global biodiversity framework aimed at halting biodiversity loss being mainstreamed in the research agenda, efforts to minimize the adverse impacts of agricultural activities on biodiversity are encouraged.

Based on this study, areas with less available evidence in Map 1 that merit future primary research include the impacts of genetic resources, natural disturbances, and water management (exposures) on all aspects of biodiversity outcomes in agroecosystems and natural ecosystems. Additionally, further research on the relationships between human and wildlife interactions, as well as chemical contaminations (outcomes) as the consequences of agricultural exposures, is also essential. Conducting research on these topics might be useful in providing a basis for future evidence.

Conversely, relationships with significant evidence in Map 1 include the impacts of land conversion on aboveground biodiversity and wild species and ecosystem functions in natural ecosystems. The other most studied relationships were crop diversification effects on aboveground biodiversity, soil biodiversity in agroecosystems, and wild species and ecosystem functions in natural ecosystems. Subsequently, a more in-depth synthesis or review study could be conducted to examine causative processes and test their significance within these themes. Additionally, conducting evidence synthesis using an approach similar to this study, but focusing on particular crop commodities, could help reveal the relationships between agriculture and biodiversity in a crop-specific fashion.

Moreover, the implications of the relatively small amount of evidence in Map 2 compared to Map 1 necessitate further primary research investigating the relationship between biodiversity that provides ecosystem services to agriculture and biodiversity that provides ecosystem disservices. The primary research topics that are recommended to be conducted are as follows: (1) the role of tropical plants as shelters, microhabitats, and litter sources for tropical agriculture production base and its management in agroecosystems; (2) the role of terrestrial invertebrates, vertebrates, and avifauna as pollinators, pest predators, and decomposers; and (3) the impact of invasive species on tropical agriculture. Moreover, since there is almost no evidence examining the relationship between wild species interactions in agroecosystems (exposure) and tropical agriculture production bases, and agroecosystem management (outcome), this topic should be prioritized. Conducting primary research on these topics, for which the evidence was lacking, might enable us to estimate the effect of biodiversity loss on crop production and not underestimate the value of ecosystem services provided by tropical biodiversity.

## Electronic supplementary material

Below is the link to the electronic supplementary material.

Additional file 1: ROSES reporting form.

Additional file 2: List of search strings used, and database sources searched.

Additional file 3: Full coded data for systematic Map 1 and Map 2.

Additional file 4: List of articles included in Map 1 and Map 2.

Additional file 5: List of studies excluded at the full-text screening with reason for exclusion.

## Data Availability

The data generated or analyzed during this study are included in this published article and its additional files.

## Footnotes

1 The visualization of the TABF schematic diagram presented here is rather different from the figure in the protocol paper since we restructured the illustration slightly to better visualize the diagram. No changes to the substantive contents of agriculture production components and biodiversity components have been made.
